# Small and mighty: adaptation of superphylum *Patescibacteria* to groundwater environment drives their genome simplicity

**DOI:** 10.1186/s40168-020-00825-w

**Published:** 2020-04-06

**Authors:** Renmao Tian, Daliang Ning, Zhili He, Ping Zhang, Sarah J. Spencer, Shuhong Gao, Weiling Shi, Linwei Wu, Ya Zhang, Yunfeng Yang, Benjamin G. Adams, Andrea M. Rocha, Brittny L. Detienne, Kenneth A. Lowe, Dominique C. Joyner, Dawn M. Klingeman, Adam P. Arkin, Matthew W. Fields, Terry C. Hazen, David A. Stahl, Eric J. Alm, Jizhong Zhou

**Affiliations:** 1 Department of Microbiology and Plant Biology, Institute for Environmental Genomics, University of Oklahoma, Norman, OK USA; 2 Biological Engineering Department, Massachusetts Institute of Technology, Cambridge, MA USA; 3 State Key Joint Laboratory of Environment Simulation and Pollution Control, School of Environment, Tsinghua University, Beijing, China; 4 Department of Civil and Environmental Engineering, University of Tennessee, Knoxville, TN USA; 5 Oak Ridge National Laboratory, Biosciences Division, Oak Ridge, TN USA; 6 Department of Bioengineering, University of California, Berkeley, CA USA; 7 Environmental Genomics and Systems Biology Division, Lawrence Berkeley National Laboratory, Berkeley, CA USA; 8 Department of Microbiology and Immunology, Montana State University, Bozeman, MT USA; 9 Department of Civil and Environmental Engineering, University of Washington, Seattle, WA USA; 10 Earth and Environmental Sciences, Lawrence Berkeley National Laboratory, Berkeley, CA USA

**Keywords:** Superphylum Patescibacteria, Candidate phylum radiation, Groundwater, Genome reduction

## Abstract

**Background:**

The newly defined superphylum Patescibacteria such as Parcubacteria (OD1) and Microgenomates (OP11) has been found to be prevalent in groundwater, sediment, lake, and other aquifer environments. Recently increasing attention has been paid to this diverse superphylum including > 20 candidate phyla (a large part of the candidate phylum radiation, CPR) because it refreshed our view of the tree of life. However, adaptive traits contributing to its prevalence are still not well known.

**Results:**

Here, we investigated the genomic features and metabolic pathways of *Patescibacteria* in groundwater through genome-resolved metagenomics analysis of > 600 Gbp sequence data. We observed that, while the members of *Patescibacteria* have reduced genomes (~ 1 Mbp) exclusively, functions essential to growth and reproduction such as genetic information processing were retained. Surprisingly, they have sharply reduced redundant and nonessential functions, including specific metabolic activities and stress response systems. The *Patescibacteria* have ultra-small cells and simplified membrane structures, including flagellar assembly, transporters, and two-component systems. Despite the lack of CRISPR viral defense, the bacteria may evade predation through deletion of common membrane phage receptors and other alternative strategies, which may explain the low representation of prophage proteins in their genomes and lack of CRISPR. By establishing the linkages between bacterial features and the groundwater environmental conditions, our results provide important insights into the functions and evolution of this CPR group.

**Conclusions:**

We found that *Patescibacteria* has streamlined many functions while acquiring advantages such as avoiding phage invasion, to adapt to the groundwater environment. The unique features of small genome size, ultra-small cell size, and lacking CRISPR of this large lineage are bringing new understandings on life of Bacteria. Our results provide important insights into the mechanisms for adaptation of the superphylum in the groundwater environments, and demonstrate a case where less is more, and small is mighty.

Video abstract.

## Introduction

Terrestrial subsurface aquifers are the repository of one-third of the Earth’s fresh water and provide an important source of water for human consumption [1]. In the USA alone, groundwater makes up 40% of the public water supply, and close to 300 million liters of groundwater are withdrawn daily [2]. Aquifers also supply water for irrigation systems, with an estimated 25% of net cultivable land irrigated via groundwater [3]. Critically, groundwater networks also interact with surface waters, participating in the exchange of water, oxygen, and both organic and inorganic nutrients [4]. The combination of human and ecological impact calls for increased understanding of groundwater geochemistry and ecology.

Groundwater microbial communities are responsible for large-scale subsurface biogeochemical cycling. Collectively, microorganisms living in groundwater cycle key elements such as carbon (C), nitrogen (N), sulfur (S), phosphorus (P), and numerous metals, and thus influence the chemical profile in subsurface and even surface aquifers [5]. Also, as novel species and genomes are being recovered from deep sequencing of subsurface environments, many new insights into microbial functions have been discovered, such as hydrogen production and fermentation of refractory sedimentary C [6]. Therefore, discovery of novel microorganisms is important to the study of microbial ecology in groundwater environments.

The newly defined superphylum *Patescibacteria* such as *Parcubacteria* (OD1) and *Microgenomates* (OP11) has been found to be prevalent in groundwater, sediment, lake, and other aquifer environments [7–9]. The superphylum was first proposed by Rinke et al. to include *Parcubacteria* (OD1), *Microgenomates* (OP11), and Gracilibacteria (GN02) which reproducibly formed a monophyletic group with data available in 2013 [10]. Later in 2015, Brown et al. acquired 796 metagenome-assembled genomes (MAGs) from groundwater samples and expanded the *Parcubacteria* and *Microgenomates* to super phyla, including 14 and 12 candidate phyla respectively [7]. Anantharaman et al. acquired 2540 MAGs from groundwater and sediment samples and proposed 47 candidate phyla in 2016 [11], with some affiliated with the *Patescibacteria*. The newly defined phyla are also referred to as candidate phylum radiation (CPR). Since then, more attention has been paid to CPR because it brings a new view of the tree of life [12–15] reconstructing a dramatically expanded version of the tree of life and revealing the dominance of bacterial diversification and the importance of uncultured bacteria from environment. However, there is little understanding of the mechanisms used by *Patescibacteria* to thrive in such a nutrient-limited water environment.

In this study, we collected samples from the Bear Creek watershed in Oak Ridge Tennessee, a site exposed to contamination from early nuclear research under the Manhattan Project. Targeting 93 contaminated and uncontaminated groundwater wells over time, we sequenced the 16S rRNA gene of 214 samples as well as the metagenomes of 12 representative wells to identify features unique to the *Patescibacteria* that may provide mechanistic understanding of their groundwater prevalence. Common potentially adaptive features include their ultra-small size, which greatly increases surface area relative to cytoplasm volume, a reduced genome coding for concentrated metabolisms and simplified membrane structures, and absence of CRISPR viral defense supplemented by alternative strategies.

## Results

### Metagenomic assembly and genome binning

The metadata including physiochemical properties and contaminant concentrations were shown in Table S1 and Figure S1, respectively. Twelve representative samples of background, low, medium, and high contamination wells were selected for shotgun metagenomic sequencing. The geochemical properties of these representative samples displayed a wide gradient of contaminant concentration in terms of heavy metal (e.g., copper, uranium and chromium) and other inorganic ions (e.g., nitrate and nitrite).

In total, 2.10 billion pair-end reads (151 bp * 2) of 636.3 Gbp from these 12 samples were obtained for metagenomic analysis (Table S2). After deduplication, quality trimming, and quality filtering, 1.37 billion high-quality paired-end reads (Q20 base: averagely 96.6% in forward reads and 91.5% in reverse reads) were used for assembly. The sequences of each sample were assembled individually. On average, 137.7 Mbp contigs (> 500 bp) from each sample was assembled with IDBA-UD, with an average N50 of 3.3 Kbp (Table S3). The maximum contig length was 1.78 Mbp from sample FW602. High-quality reads of the individual samples were mapped to the assemblies to calculate read coverage of contigs in each sample. The average alignment rate (reads to contigs of the same sample) was 29.8% with the range from 3.4 to 73.1% (Table S4).

Contigs were then clustered with MetaBat based on their coverage pattern and tetranucleotide frequency, resulting in 526 bins. After quality evaluation with CheckM, 158 genome bins with completeness > 70% and contamination < 10% were selected for further analysis and they had an average completeness of 90.4%, and average contamination of 2.5%. The average genome size was 3.38 Mbp, with an average maximum contig length of 200 Kbp and an average N50 of 90 Kbp (Table S5).

### Small genome size of the Patescibacteria superphylum

**Fig. 1:**
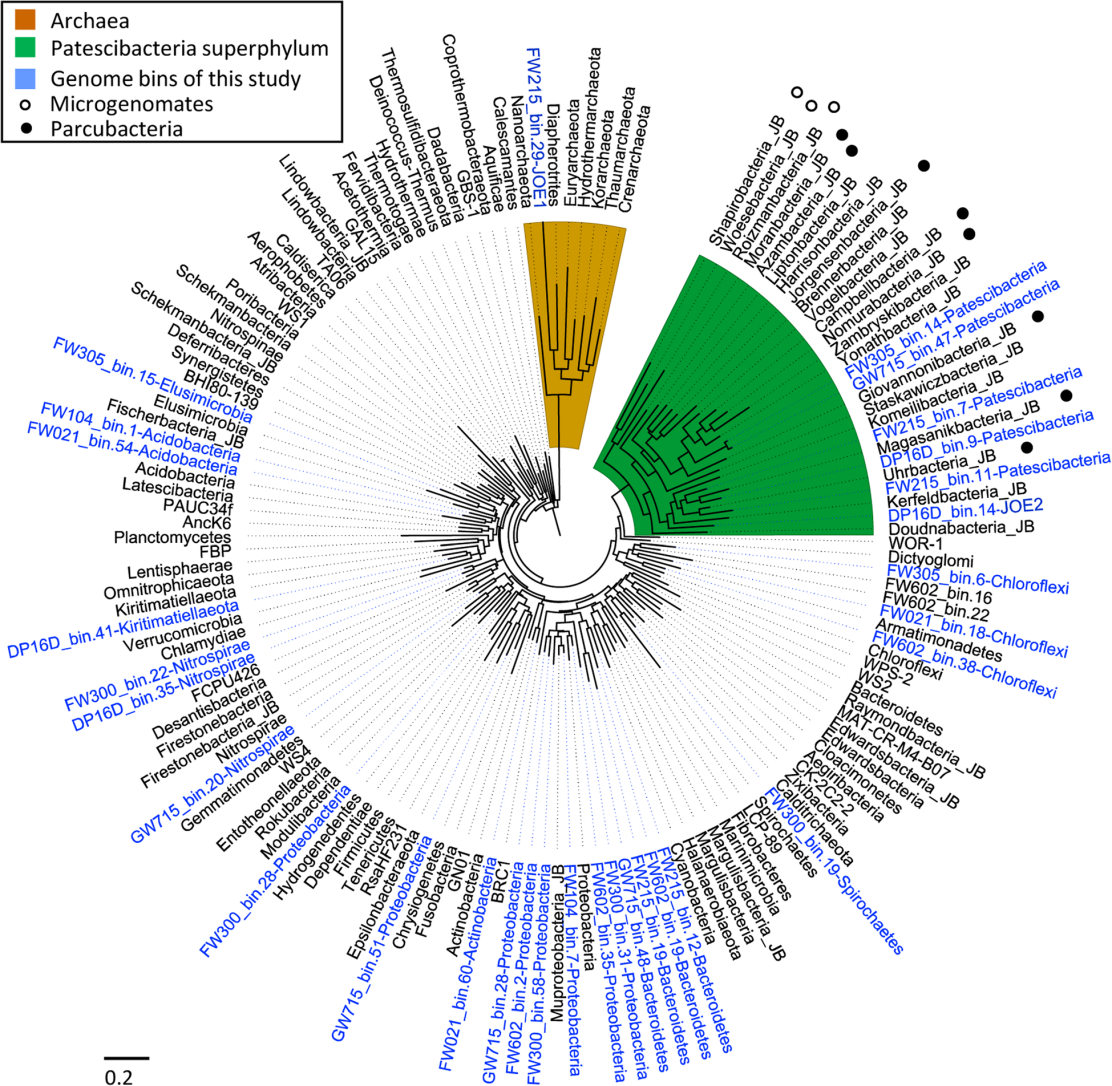
Maximum likelihood (ML) tree of well-established bacterial and archaeal phyla, and *Patescibacteria* superphylum based on full-length 16S rRNA genes from complete genomes or genome bins (with 16S rRNA gene > 1300 bp, completeness > 70%, and contamination < 10%). The suffix JB indicate phyla proposed by Banfield group. The open and closed circle indicates the superphylum *Microgenomates* and *Parcubacteria* proposed in the study of Banfield group. Because we applied the limitation of 16S rRNA gene length, genome completeness and contamination, not all phyla of *Microgenomates* and *Parcubacteria* are represented

We collected nearly full-length 16S rRNA genes from genomes of well-established phyla, genome bins of newly defined phyla from Banfield group [7, 11, 13], and from our own to construct a maximum likelihood (ML) tree (only phylum representatives with 16S rRNA genes > 1300 bp, completeness > 70%, and contamination < 10% were considered, see Table S6). Interestingly, there was a large independent clade with long branch lengths, including 20 newly defined candidate phyla from Banfield group (some were also referred to as superphylum *Parcubacteria* and *Microgenomates* in the study of Christopher Brown et al. [7] together with six genome bins assembled as part of this study) (Fig. 1, see the clade in green). The phylogeny of concatenated RP16 genes also supports the independent lineage (Figure S2). Based on the previous proposal of superphylum *Patescibacteria* [9], the classification of the latest SILVA database (version 132) [16], and the taxonomic structure of recently organized genome-based database GTDB [17], we refer to this clade as the *Patescibacteria* superphylum in this study.

We calculated the calibrated genome sizes (genome size/completeness) of newly defined phyla and genome bins from this study (only genome bins with completeness > 70% and contamination < 10% were considered) and compared them to the genome sizes of the well-established phyla collected from NCBI (Table S6). Interestingly, the *Patescibacteria* superphylum has a genome size of 1.1 ± 0.2 Mbp compared to 3.5 ± 1.5 Mbp for the other well-established bacterial phyla. This result indicated highly reduced genome sizes of the superphylum which is consistent to previous findings [7].

We used the following criteria to propose phylum-level lineages: (i) full-length 16S rRNA gene share < 75% similarity to established phyla; (ii) phylogenetic location based on 16S rRNA gene is consistent to that based on rp16 concatenated ribosomal proteins at subclade level; and (iii) availability of a complete or draftgenome sequence with completeness > 70% and contamination < 10%. As a result, two genome bins were proposed as candidate division JOE1 and JOE2 respectively. Because we currently only have one genome for each and thus cannot confirm monophyletic topology of them, we propose them as candidate division rather than candidate phyla. Phylogenetic trees of both full-length 16S rRNA gene and concatenated rp16 genes (Figure S2) showed that the candidate division JOE2, together with five other genome bins, were members of the *Patescibacteria* superphylum (Fig. 1). Candidate division JOE1 defined a new lineage within the Archaea.

### The Patescibacteria superphylum has retained the essential function such as genetic information processing despite reduced genomes

We compared our *Patescibacteria* genomes and 20 representative genomes of *Patescibacteria* phyla from Banfield group to other well-established bacterial genomes to gain insights into the unique functional features and metabolism of the *Patescibacteria* superphylum. The selected genome bins (completeness > 70%, contamination < 10%, 16S rRNA gene > 1300 bp) of the *Patescibacteria* superphylum had a completeness of 75.0 ± 5.6% and contamination of 1.6 ± 2.2% (Figure S3A), and they had 19.4 ± 1.0 unique tRNA genes (Figure S3B), which indicated that these genome bins are qualified for genomic comparison.

**Fig. 2:**
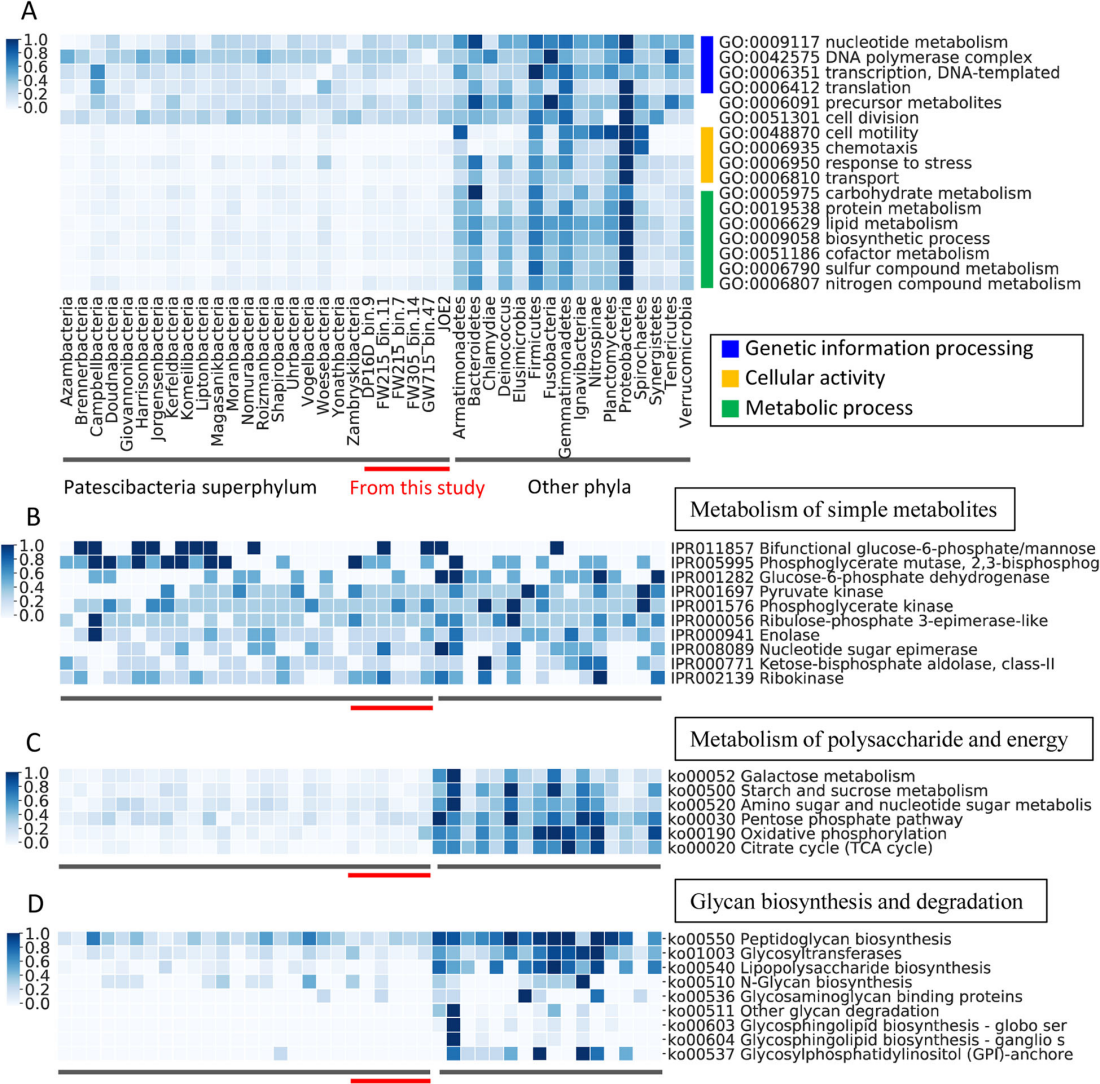
Comparison of functions and metabolisms between the superphylum *Patescibacteria* and the other phyla. **a** Shows the comparison of biological process including genetic information processing, cellular activity and metabolic process at level 2 of GO term classification system. **b** Shows the comparison of basic simple intermediate metabolites at the level 3 of Interpro2Go classification. **c** Shows the comparison of polysaccharide metabolism and energy at level 3 of KEGG classification. **d** Shows the comparison of glycan biosynthesis and degradation at level 3 of KEGG classification. The color represents gene numbers which were linearly standardized [(X-min)/max] to 0-1 for each row

Despite small genome size, the *Patescibacteria* genome bins of this study have retained functional genes involved in genetic information processing, including nucleotide metabolic processes (synthesis and degradation), DNA polymerase complex, DNA-templated transcription and translation, which is similar to the non-*Patescibacteria* phyla (Fig. 2a). There are 53 ± 2 genes of these functions in the six genomes, which is consistent to the whole superphylum *Patescibacteria* (55 ± 6). The other phyla have slightly higher number of these genes (69 ± 13), indicating a retainment of most of these genes in *Patescibacteria*. Functions of genetic information processing are conserved among phyla in spite of the reduced genome size of *Patescibacteria* superphylum, especially the genes coding for ribosomal proteins (Figure S4). The *Patescibacteria* superphylum has 50 ± 5 genes for small and large subunit ribosomal proteins which is close to other phyla (58 ± 11 genes), and most of them are core ribosomal proteins among all phyla. Interestingly, the *Patescibacteria* superphylum exclusively lack ribosomal protein L30 while the other phyla generally have it, which is consistent with previous findings [7]. In addition, we found that ribosomal protein L36 was also missing in 20 of the 26 phyla of *Patescibacteria* (Figure S4). Other important functions including key intermediate metabolites (such as pyruvate) and cell division are also not significantly reduced in the superphylum *Patescibacteria*. These results indicated that the newly found *Patescibacteria* superphylum had retained essential functions such as genetic information processing which is important to growth and reproduction despite significantly reduced genomes.

### The Patescibacteria superphylum has reduced cellular activities and metabolic potentials

Functions related to cellular activities, however, have been significantly streamlined in the genomes of superphylum *Patescibacteria* (Fig. 2a). In the *Patescibacteria* genomes of this study, the functions of cell motility (including flagellar components, flagellin and flagellar motor proteins) and chemotaxis (including chemoreceptor glutamine deamidase, chemotaxis methyl-accepting receptor, and signal transduction response regulator) are comprised of 0.8 ± 1.2 genes which is consistent to the whole superphylum *Patescibacteria* (0.7 ± 1.2), but the other phyla have 17 ± 18 genes (Welch’s *T* test, *P* < 0.001). The *Patescibacteria* genomes of this study have 20 ± 1 functional genes involved in response to stresses, including heat shock proteins, cold shock proteins, osmotic stress-related proteins, and regulator proteins, which is consistent to the whole superphylum (21 ± 6) but the other phyla have 51 ± 22 genes (Welch’s *T* test, *P* < 0.001). In terms of substance transport (including transport proteins, permeases, secretion proteins, and ABC transporter), our genomes have 36 ± 5 and the whole superphylum *Patescibacteria* has 39 ± 12 genes involved but it is 253 ± 170 in the other phyla (Welch’s *T* test, *P* < 0.001).

In terms of metabolisms, the superphylum *Patescibacteria* has reduced redundant functions involved in carbohydrate, protein, and lipid metabolisms; biosynthetic process, cofactor metabolism; and sulfur and nitrogen compound metabolisms (Figure 2a). The *Patescibacteria* genomes of this study have 18 ± 5 genes and the whole superphylum has 17 ± 5 genes involved in carbohydrate metabolism, but the other phyla have 66 ± 39 genes (Welch’s *T* test, *P* < 0.001). Interestingly, among the carbohydrate metabolism genes, the *Patescibacteria* superphylum has retained genes for basic metabolisms of simple intermediate metabolites such as pyruvate, phosphoglycerate, and monosaccharide including glucose, ribulose-phosphate, ribose, and ketose (Fig. 2b). However, the genes for sugar derivatives (such as amino sugar and nucleotide sugar) and polysaccharides (such as galactose, starch, and sucrose) have been sharply reduced in *Patescibacteria* superphylum (Fig. 2c). In terms of aerobic respiration, the *Patescibacteria* superphylum lack genes for citrate cycle and oxidative phosphorylation, except the ATPase complex (Fig. 2c), which is consistent to the previous results [7], suggesting an adaptation to anaerobic environment in groundwater.

In terms of biosynthetic process, our *Patescibacteria* genomes have only 68 ± 5 genes and the whole superphylum has 58 ± 19 genes involved, but the other phyla have 249 ± 110 genes (Welch’s *T* test, *P* < 0.001, Fig. 2a). In the process of glycan biosynthesis and metabolism, the *Patescibacteria* superphylum retained functional genes for synthesis of peptidoglycan and lipopolysaccharide (Fig. 2d) which are related to essential functions of bacterial cell wall and outer membrane respectively. However, genes for biosynthesis and degradation of other glycans are almost missing in the *Patescibacteria* superphylum.

### The Patescibacteria superphylum has simplified membrane structures

Previous studies have investigated the ultra-small cell sizes of *Microgenomates* (OP11), *Parcubacteria* (OD1) belonging to *Patescibacteria* [7–9]. Here, we confirm the small cell size of the whole *Patescibacteria* superphylum by comparing the relative abundances of the total OTUs of *Patescibacteria* captured on 0.2-, 3-, and 10-μm filters.

For microbial community composition study, 214 samples from 93 wells across a broad geochemical gradient (in terms of pH, DIC, DOC, DO, and contaminant concentrations, etc., Figure S1) were analyzed. After paired-end sequence merging and demultiplexing, 13,397,875 reads were recovered for all the samples. Chimeric sequences (53,114) and singletons (2,261,068) were removed. In total, 33,133 operational taxonomic units (OTUs, 97% identity) were acquired, and 12 classified as Chloroplast were removed. Rarefaction showed that most curves had reached a plateau (Figure S5), indicating that the sequencing depths were sufficient to represent the diversity and composition of the microbial communities. In total, 192 samples with multiple filtration fractions were compared. As a result, we found that the *Patescibacteria* cells were highly enriched (Wilcoxon signed-rank test, *P* < 0.001) in the small-size filters (0.2 μm) by averagely 15.7 times compared to the larger pore-size filters (3 or 10 μm, Figure S6), which indicates a smaller cell size of *Patescibacteria* than other phyla.

**Fig. 3:**
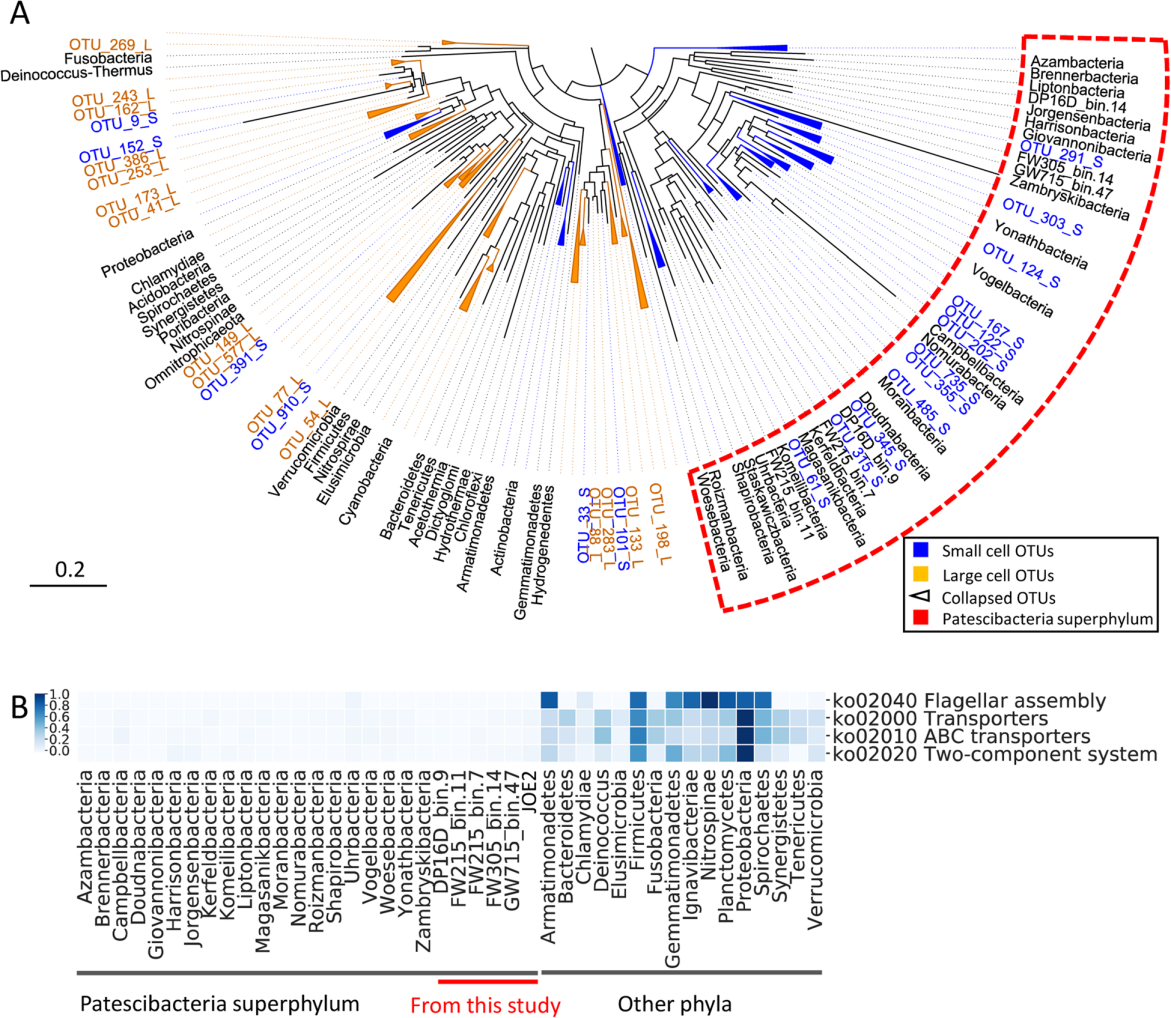
Small cell size of *Patescibacteria* superphylum and the simplified membrane structure. **a** Shows the maximum likelihood (ML) tree of the *Patescibacteria* superphylum and small- and large-cell OTUs (captured on 0.2- and 0.4-μm filters respectively) with other well-established bacterial phyla as references. **b** Shows the simplified membrane structures of *Patescibacteria* superphylum in comparison to the other phyla, including flagellum, transporters and two-component system. The color represents gene numbers which were linearly standardized [(X-min)/max] to 0-1 for each row

To further investigate the feature of small cell size in terms of phylogeny, we collected 95 large-cell OTUs and 70 small-cell OTUs that appear exclusively in large bacteria (captured in 0.4 μm filter) and small bacteria (passing 0.4 μm filter and captured in 0.2 μm filter) respectively from water samples (groundwater, river, lake, etc.) [9]. These small bacteria were also shown to have low nucleic acid-content (LNA) by flow cytometry, namely small-genome bacteria. We constructed a phylogenetic tree of these OTUs and the *Patescibacteria* superphylum, with representative well-established bacteria phyla as references. Interestingly, no large-cell OTUs appeared in the *Patescibacteria* superphylum, and most of the small-cell OTUs (54/70) were scattered in the superphylum (Fig. 3a). Most of the small-cell OTUs in the superphylum (50/54) were classified as *Patescibacteria* at phylum level, and the others were unclassified. These results infer that the superphylum *Patescibacteria* has ultra-small cell size, which is consistent to the previous findings where ultra-small bacteria cells were enriched by filters and identified as members of this superphylum [7–9].

A lack of genes for flagellar assembly and chemotaxis functions among the new *Patescibacteria* genomes and the whole superphylum *Patescibacteria* (Figs. 2a and 3b) suggest that the superphylum *Patescibacteria* is nonmotile. An absence of chemotactic motility is also consistent with a reduction in two-component systems responsible for sensing external environmental change (~ 5 in the *Patescibacteria*) compared to other phyla, which suggests that motility is a luxury in the relatively stable subsurface microenvironment. The other phyla have 200 ± 122 transporter proteins and 63 ± 47 ABC transporter proteins while the superphylum *Patescibacteria* has only 13 ± 7 and 3 ± 3 respectively (Welch’s *T* test, *P* < 0.001, both functions). The superphylum *Patescibacteria* only retains essential transporters for basic substance transport (Figure S7), such as the multiple sugar transport system substrate-binding protein (K02027), cation H^+^ antiporter (K07301), zinc and cadmium transporter (K16267), magnesium transporter (K03284), and lactate permease (K03303). The reduced number of transporters together with the reduced carbohydrate metabolisms (Fig. 2a) suggest a simplified metabolism for energy and C utilization. The highly simplified membrane structure of *Patescibacteria* may contribute to the small cell size of it.

### The Patescibacteria superphylum lacks CRISPR, but might have alternative strategy and thus is less invaded by phage

**Fig. 4:**
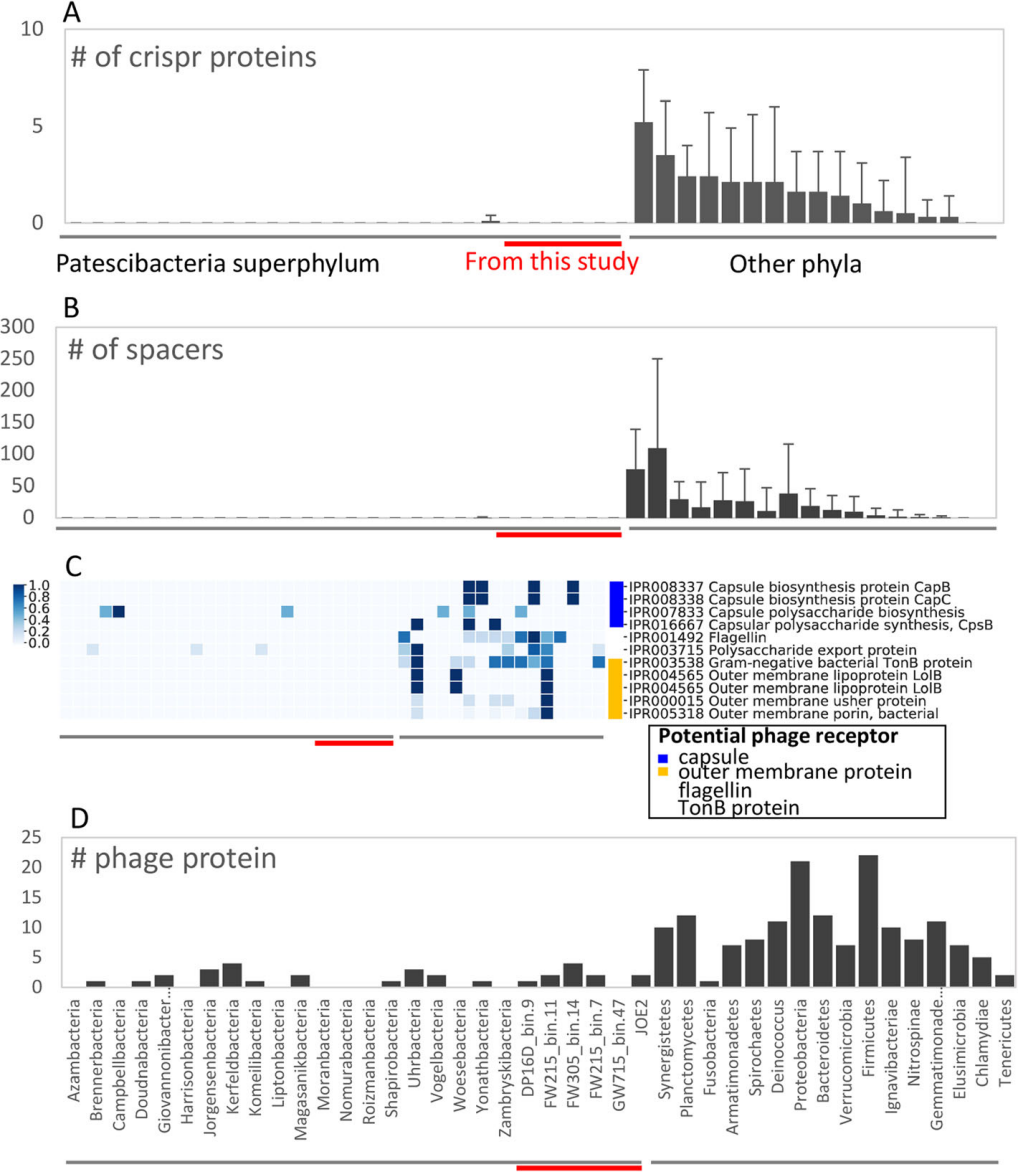
Comparison of CRISPR proteins (**a**), CRISPR spacers (**b**), potential phage receptors (**c**) the color represents gene numbers which were linearly standardized for each row [(X-min)/max] to 0-1), and phage proteins (**d**) between the *Patescibacteria* superphylum and the other well-established phyla. The error bars represent the standard deviation of each phylum

For comparison of CRISPR sequences and the associated proteins, we collected 502 complete or draft genomes of the non-*Patescibacteria* phyla used in the above genomic comparison (~ 31 genomes per phylum) from NCBI and compared the CRISPR spacer number (indicating the capacity of phage defense) and CRISPR-associated protein (cas) number to our *Patescibacteria* genomes (six genomes) and *Patescibacteria* genomes from Banfield group (114 genomes with completeness > 70% and contamination < 10%, 16S rRNA gene > 400 bp). The non-*Patescibacteria* phyla has on average 23.9 CRISPR spacers (maximum 109.1 ± 140.7 in Planctomycetes, Fig. 4a) and 1.7 cas proteins (maximum 5.2 ± 2.7 in Synergistia, Fig. 4b). However, the 120 genomes of *Patescibacteria* have no detectable CRISPR sequence and cas protein except one Zambryskibacteria genome bin which has four CRISPR spacers and one cas protein (Fig. 4a, b). The CRISPR spacer and cas protein are highly positively correlated (Pearson correlation coefficient: 0.88, *P* < 0.001), and CIRSPR sequences are absent wherever there are no cas proteins. We also analyzed non-*Patescibacteria* draft genomes published by the Banfield group and in our binning (Figure S8, S9) and found on average 11.1 CRISPR spacers and 2.2 cas proteins in the 31 non-*Patescibacteria* genome bins of Banfield group, and 27.5 CRISPR spacers and 1.9 cas proteins in the 26 non-*Patescibacteria* genome bins of this study, excluding the possibility that fragmentation of the draft genomes invalidates the identification of CRISPR sequence. These results are similar to the findings of previous reports [18].

The lack of CRISPR-mediated phage defense suggests that the *Patescibacteria* superphylum could have alternative strategies to resist phage infection, given that that bacteria is prevalent in water environments. We hypothesize that the bacteria might block phage from attaching to the cell membrane because of the simplified membrane structures (Fig. 3b). Therefore, we compared the superphylum *Patescibacteria* and the other phyla in terms of phage receptor on cell membrane. As a result, the potential phage receptors detected including proteins related to capsule, outer membrane proteins, flagellin, and TonB protein are generally missing in the superphylum *Patescibacteria* (averagely 0.3 genes) in comparison to the other phyla (8 ± 9 genes, Fig. 4d). The absence of potential phage receptors in the membrane may serve as an alternative strategy of *Patescibacteria* to resist phage invasion.

Although the *Patescibacteria* superphylum lacks CRISPR-mediated viral defense, considered the most important mechanism of bacterial immunity, interestingly, it seems not sensitive to phage infection due to lack of phage receptors. The functional genes of phage and prophage were much less present in *Patescibacteria* genomes compared to the other phyla. We compared representative genomes *Patescibacteria* and other phyla in terms of phage-associated proteins (including phage structure proteins, phage integration proteins, regulators, etc., Figure S10). The *Patescibacteria* genomes of this study has 1.8 ± 1.3 phage-associated proteins per cell and the whole superphylum has 1.2 ± 1.3, but the other phyla contain 9.6 ± 5.6 proteins, which is eight times as high as *Patescibacteria* phyla (Fig. 4c, Welch’s *T* test, *P* < 0.001). The phage-associated proteins detected in the bacterial genomes are mainly acquired from temperate phages which are integrated into the genomes as prophages. The number of prophage proteins indicate the extent to which the bacteria have been invaded by phages. Our results showed that the *Patescibacteria* have been less subjected to phage invasion than other phyla.

## Discussion

In our study, we acquired 158 good-quality MAGs from groundwater samples and analyzed them with MAGs of previous studies. We found that the *Patescibacteria* superphylum has highly reduced redundant functions of metabolisms, cellular activities, and stress response, while retaining the essential functions such as genetic information processing. The *Patescibacteria* has ultra-small cell sizes and simplified membrane structures including diminished flagellar assembly, transporters, and two-component systems. Although the bacteria lack CRISPR, which is important for phage defense, they could have alternative strategies to resist phage infection.

Previously, Brown et al. proposed 26 candidate phyla from groundwater samples belonging to the super phyla *Parcubacteria* and *Microgenomates* [7]. They discovered certain usual features including ultra-small cell sizes, insertions inside 16S rRNA genes, missing ribosomal proteins L30, small genome sizes, and metabolic limitations. The *Parcubacteria* and *Microgenomates* belong to the super phyla *Patescibacteria* and account for about a half of the super phyla in our study (Fig. 1). We here conducted a more extensive genomic comparison by including the additional sequences recovered from Oak Ridge. In general, members of the *Patescibacteria* superphylum have retained basic metabolic functions centered on glucose and pyruvate, and lost numerous functions related to motility, chemotaxis, outer membrane function, polysaccharide metabolism, biosynthesis, and nutrient transport. They have retained basic systems for gene expression and replication, especially the surprisingly conserved ribosomal proteins despite highly reduced genome size.

Anantharaman et al. proposed 47 candidate phyla from groundwater and sediment samples through metagenomic binning [11] and they found the interactions of microorganisms in terms of biogeochemical processes such as nitrogen cycle. Through phylogenetic analysis and genomic comparison, we found that ten of the 47 candidate phyla belong to the *Patescibacteria* because they form an independent lineage with the super phyla *Parcubacteria* and *Microgenomates* as well as MAGs of this study consistently based on both nearly full-length 16S rRNA gene and concatenated RP16 genes. In addition, similar to the other *Patescibacteria* phyla, they also share the features of reduced non-essential functions and metabolisms and lack of CRISPR among others.

In addition to the bacterial cell size study of Brown et al. [7], Luef et al. also proved the ultra-small cell size of *Microgenomates* (OP11), *Parcubacteria* (OD1), and Katanobacteria (WWE3) from groundwater using cryogenic transmission election microscope [8]. Their cell size was about 0.009 ± 0.002 μm^3^_._ These small cells do not have outer membrane and are inferred to be gram-positive bacteria. They also found pili-like structures of these ultra-small microorganisms and inferred inter-organism substance exchange through it. The electron microscope images showed no flagellum and outer membrane of *Microgenomates* and *Parcubacteria*, which is consistent to our genomic results. In our study, we found that the *Patescibacteria* superphylum has reduced functions of cell motility and flagellum, outer membrane, polysaccharide metabolism, biosynthesis process, transporter for nutrient uptake, and retained metabolisms of simple metabolites such as glucose and pyruvate. The bacteria may use the pili-like structure for nutrient transport from hosts (could be either bacteria or protists), because they have reduced functions of biosynthesis and transporter for nutrient uptake according to our genomic comparison.

**Fig. 5:**
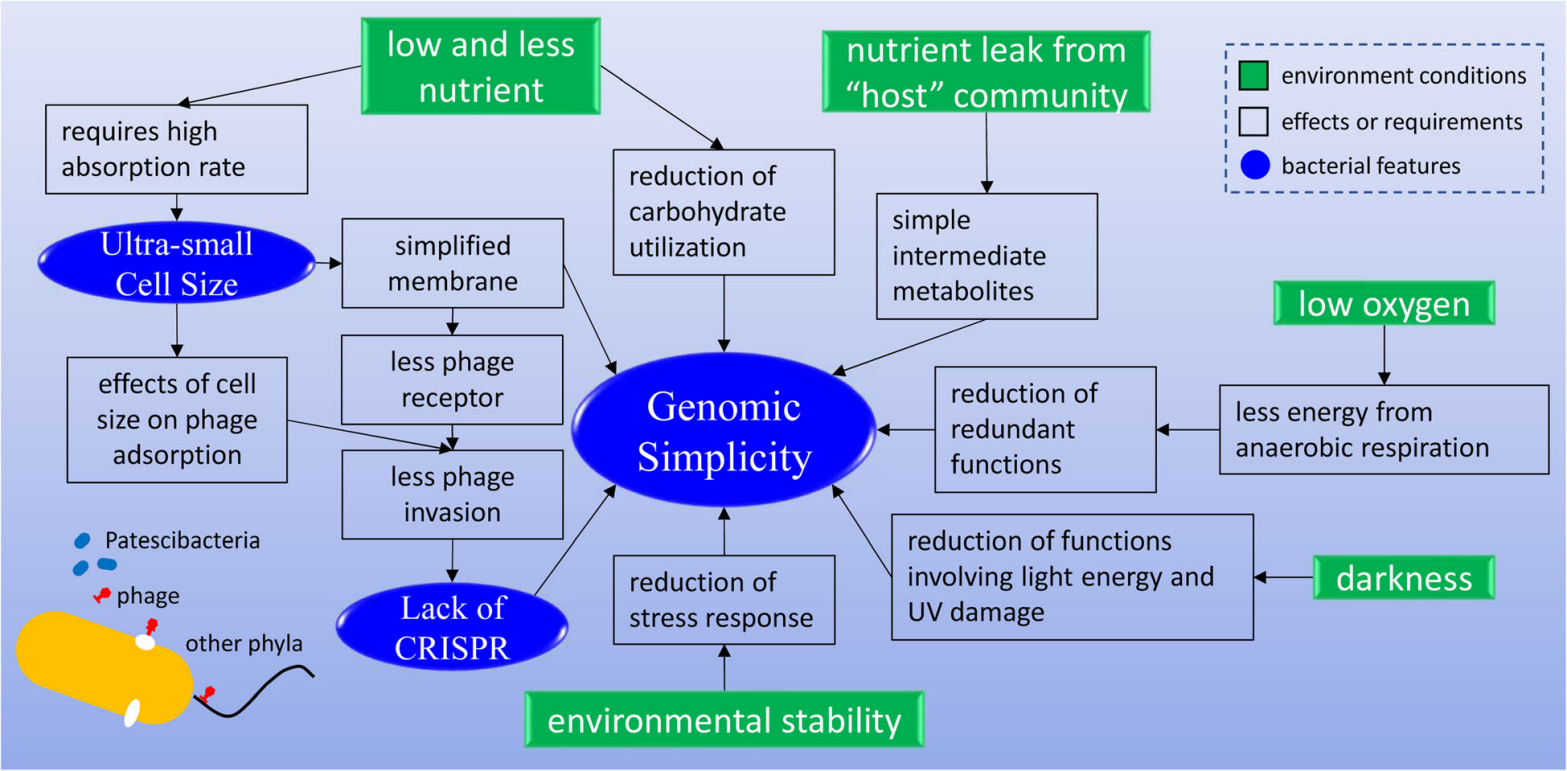
Linking the environmental conditions of groundwater to the *Patescibacteria* features of reduced genome, lack of CRISPR viral defense, and ultra-small cell size

Based on environmental condition of groundwater and the metabolic and functional features of *Patescibacteria*, we proposed that the adaptation of *Patescibacteria* to groundwater environments facilitates the features of small genome size, lack of CRISPR viral defense, and ultra-small cell size (Fig. 5) as below.

The groundwater is an environment with nutrients (including C, N, S, and P) in low concentration and low diversity (e.g., in comparison to soil). The low and less nutrients may have reduced the metabolic capacity of *Patescibacteria* and thus the carbohydrate utilization genes for polysaccharides, disaccharides, and amino sugar among others were streamlined in the genomes. Chemoautotrophic metabolisms (such as sulfur oxidation, ammonia oxidation, and nitrite oxidation) were not detected either. Some of the *Patescibacteria* may rely on hosts such as larger bacteria or protist (*Parcubacteria* [19] for nutrients supply (they have pili-like structures [8] for nutrient uptake). According to the Black Queen Hypothesis [20], bacteria with reduced genomes may rely on bacterial community with full metabolism capacity for “public goods.” The *Patescibacteria* with highly reduced genomes may rely on simple intermediate metabolites from “host” community for energy because the metabolic pathways for simple metabolites were retained in the genomes, which facilitates the genomic simplicity.

The environment of low nutrient concentration requires bacteria to increase absorption rate. The *Patescibacteria* seem to have adopted the strategy of shrinking cell size (~ 0.3 μm). Small cell size has been proved to increase metabolic rate [21] because smaller cells have a higher ratio of surface area to volume, which speeds up the substance exchange across cell membrane. Cell membrane of *Patescibacteria* was simplified because of cell size reduction. As a result, the reductions of some membrane structures, such as flagellum, capsule, and outer membrane protein which could be taken advantage by phage as receptors [22, 23], in turn make the bacteria escape from phage attachment, thus the bacteria are less invaded by phage (indicated by the less phage-associated proteins detected in genomes). Because phage receptors such as flagellin and capsule proteins have only been identified in model phages, the role of these proteins as phage receptors in *Patescibacteria* still needs to be verified.

Small cell size could serve as a merit to escape from phage adsorption. There are studies in the effects of bacterial cell size on the phage adsorption and burst. Hilla Hadas et al. [24] found that adsorption rate of T4 phage was positively correlated to *Escherichia coli* cell size, suggesting that smaller cell size prevents phages from attaching. Charles Choi et al. [25] investigated the effect of cell size of *E*. *coli* B23 on the T4 phage burst size and found that larger cell has increased phage burst. Moreover, physically, ultra-small cell size makes it hard for phages to target, and also there is no sufficient space for the phages (~ 0.2 μm) to attach. These alternative strategies might have complemented the lack of CRISPR which plays important roles of viral defense in other phyla but has been deleted in *Patescibacteria*. The lack of CRISPR and reduced phage receptors are not considered evidences of the resistance to phages, but they could be taken as the effects of any possible phage resistance, because resistance to phage would cause reduced CRISPR to save genetic materials and energy, reduced phage receptors on membrane to save space for other membrane proteins due to reduced surface area, and less prophage proteins.

Groundwater has a low oxygen concentration [26] because it is underground and there is no light for plankton to generate oxygen through photosynthesis. The 93 wells of this study had a dissolved oxygen (DO) concentration of 1.1 ± 1.7 mg/L (Table S1) and light intensity is considered zero in the natural groundwater. Because of the low oxygen concentration, *Patescibacteria* only rely on anaerobic respiration (lacking oxidative phosphorylation pathway) which provides less energy than aerobic respiration. Functions requiring much energy such as flagellar motility and secondary metabolisms were thus reduced in *Patescibacteria*. Dark environment also reduces functions involving light energy. There were no photosynthetic pathways and corresponding CO_2_ fixation pathways detected in *Patescibacteria* genomes. Dark environment also reduces functions of light repair of DNA damage. Without ultraviolet (UV) radiation, functions involved in UV stress were also reduced in *Patescibacteria* (data not shown). The anaerobic and dark environment and all these effects contributed to the reduced genome size of *Patescibacteria*.

Stability of environmental conditions may also have contributed to reduced genome size of *Patescibacteria*. The uncontaminated wells of this study had very stable conditions such as temperature (16.6 ± 3.9 °C, Table S1) and pH (6.5 ± 0.7, Table S1). Less variability of environment was demonstrated to select bacteria with smaller genomes [27]. The stable physical conditions such as temperature and pH allow *Patescibacteria* to survive without investing in an adaptive response to environmental perturbation. This is verified by the fact that *Patescibacteria* was sensitive to contamination probably because of the reduced metabolic potential and stress response due to genome reduction.

## Conclusions

In summary, *Patescibacteria* constitutes a large part of the updated tree of life and is prevalent in water environments. We found that *Patescibacteria* has streamlined many functions while acquiring advantages such as avoiding phage invasion, to adapt to the groundwater environment. The unique features of small genome size, ultra-small cell size, and lacking CRISPR of this large lineage are bringing new understandings on life of Bacteria. Our results provide important insights into the mechanisms for adaptation of the superphylum in the groundwater environments, and demonstrate a case where less is more, and small is mighty.

## Materials and methods

### Sample collection and environmental conditions

Groundwater samples were collected from contaminated areas (243 acres) and uncontaminated background areas (402 acres) of the Department of Energy’s Oak Ridge Field Research Center (ORFRC) in Bear Creek Valley watershed of Oak Ridge, Tennessee, US (http://www.esd.ornl.gov/orifrc/). The contamination source is the waste disposal ponds of S-3 located within Y-12 national security complex, including radionuclides, nitrate, sulfide, etc. In 1988, the S-3 ponds were shut down and capped. However, contaminants leached out and became a groundwater contaminant plume across the field site. Concentration of contaminants including the radioactive uranium and other metals (such as Cr, Cu, and Cd), inorganic ions (such as nitrate, nitrite, and sulfate), and chemical characteristics of temperature, pH, redox potential, dissolved organic/inorganic carbon were measured. In total, 93 wells were sampled using filters of different sizes (10, 3, and 0.2 μm) from November 2012 to February 2013. The detailed sampling process was described in Smith et al [28]. For shotgun metagenomic sequencing, 12 wells of different contamination degree were selected to filter 6–10 L water with 0.2-μm filters from May to August 2014. Filter samples were kept on ice and transported to laboratory before further processes.

### DNA extraction for amplicon sequencing and metagenomic sequencing

The process of DNA extraction and PCR amplification were described in Smith et al. [28]. Briefly, the filtered microbial cells were lysed with 1.5 ml phosphate buffer and an equal volume of Miller SDS lysis buffer [29]. Phenol-chloroform-isoamyl alcohol (25:24:1) and chloroform were used to denature protein. After centrifugation, the supernatant was purified through a spin column according the manufacturer’s instruction.

### PCR amplification of 16S rRNA gene, sequencing, and processing

We used phasing amplicon sequencing (PAS) approach [30] to sequence the V4 region of 16S rRNA genes of Bacteria and Archaea. Equal amount of product of the samples were pooled and purified with a QIAquick gel extraction kit (QIAGEN Sciences). Library of the 16S rRNA gene amplicon sequencing were constructed using MiSeq reagent kit (Illumina, San Diego, CA) following the manufacturer’s instruction, and DNA was sequenced using an Illumina MiSeq platform.

The amplicon sequence data was analyzed using an internal pipeline (ASAP, http://zhoulab5.rccc.ou.edu/pipelines/ASAP_web/job_submission.php). The quality of MiSeq sequences (2 * 251 bp) were evaluated with FastQC (version 0.11.5). The pair-end sequences were merged using PEAR (version 0.9.10) [31] based on the 3’ overlap with a quality score cutoff of 20 and minimum overlap length of 40 bp. Reads were assigned to samples (demultiplexing) using the program split_libraries_fastq.py of QIIME packages (version 1.9.1) [32] based on the barcodes with the maximum barcode error of 0 and trimming quality score cutoff of 20. Primer sequences (forward and reverse) were trimmed with zero mismatch allowed. Multiple sequencing rounds (two rounds in this study) were combined after library splitting. Dereplication was performed using VSEARCH (version 2.4.4) [33] with the command—derep_fulllength (with the option of -sizeout for sequence abundance output). Operational taxonomic units (OTUs) were clustered using UPARSE (command -cluster_otus of USEARCH) [34] with OTU identity threshold of 0.97 and singletons and chimeric sequences were removed during this process. OTU table was made using command of -usearch_global of VSEARCH. The representative sequences of OTUs were classified using SINTAX [35] with the database SILVA (version 132, April 2018) and confidence cutoff of 0.9.

### Metagenomic sequencing, assembly, and genomic binning

Twelve representative samples for background, low, median, and high contamination wells were selected for metagenomic sequencing. Libraries were constructed using genomic DNA with KAPA Hyper Prep Kit (KR0961) following the manufacturer’s instruction, and DNA was sequenced using an Illumina HiSeq 3000 platform.

The metagenomic data was processed using an internal metagenomic pipeline (ARMAP, http://zhoulab5.rccc.ou.edu/pipelines/ARMAP_web/job_submission.php). The sequence data was first subjected to quality evaluation using FastQC to check the quality profile, duplication rates, and contamination rates. CD-HIT (version 4.6.8) [36] was then used to remove duplicates with identity cutoff of 100%. NGS QC Toolkit (version 2.3.3) [37] was used for quality trimming and filtering. Reads with residual adaptors were filtered out based on paired-end adaptor library. Poor-quality bases with quality score < 20 were trimmed from 3′ end until the first base with quality score ≥ 20. Trimmed reads with length of > 120 were further filtered with average score cutoff of 20. Reads with > 1 ambiguous bases were removed.

The pair-end reads (fasta) of each sample after quality trimming and filtering were assembled using IDBA-UD (version 1.1.1) [38] with minimum kmer value of 31, maximum kmer value of 121, and step size of 10 (--mink 31 --maxk 121 --step 10). Pre-correction with maximum mismatch of 3 was performed before assembly (--max_mismatch 3 --pre_correction). Large memory was required for some samples with complex community composition and we ran the assembly in a supercomputer with 1 Tbp memory and 80 CPU cores. The contig sequences were imported into NGS QC Toolkit (version 2.3.3) [37] for calculation of contig length profile (N50Stat.pl). The individual assemblies of the 12 samples were merged using PCAP [39] with adjusted overlap score cutoff of 4500 (-s 4500) and overlap percent identity cutoff of 95% (-t 95). The merged assembly was summarized using N50Stat.pl of NGS QC Toolkit.

Pair-end reads of the 12 samples after quality trimming and filtering were mapped to the assembled contigs using Bowtie2 (version 2.2.9) [40] using global alignment (--end-to-end) and the preset of sensitive alignment (--sensitive). The output sam files were converted into bam files which were then sorted using SAMtools (version 1.3.1) [41] with command of “view.” Duplicate reads were further removed using command “rmdup” (reference-based method) of SAMtools. The output bam files were further sorted and indexed. Coverages of contigs in each sample were calculated using program “jgi_summarize_bam_contig_depths” of the MetaBat (version 0.32.4) [42] package with the identity cutoff of alignment 0.97. The coverage output and the contig sequence file were then imported into MetaBat for genome binning analysis based on clustering of contig coverages in the 12 samples and sequence composition (parameter: -m 1500–B 20 --superspecific). The bins were evaluated in terms of completeness and contamination using CheckM (version 1.0.5) [43]. Genome bins with completeness > 70% and contamination < 10% were further selected for analysis.

### Functional annotation of genomes and genome bins

Genomic annotation and comparison were performed using an internal pipeline (Automatic Genomic Analysis Pipeline, AGAP). In the pipeline, genomic annotation was conducted using PROKKA (version 1.11) [44]. First, the finished genomes and draft genomes were subjected to gene calling using Prodigal (version 2.6) [45] with output of translated protein sequences, single mode, and genetic code of Bacteria and Archaea (-a, -p single, -g 11). The protein sequences were used for completeness and contamination evaluation with CheckM (version) [43]. Then rRNA genes were predicted using Barrnap (version 0.7) of PROKKA. Pseudogenes and coding sequences overlapping with tRNA and rRNA gene were removed by PROKKA. The 16S rRNA genes were used for taxonomic classification using RDP Classifier (version 2.12) [46]. Protein sequences were subjected to DIAMOND (version 0.8.5) [47] search (BLASTp) against NCBI NR database (Jan 2016) with *E* value cutoff of 1e-5, coverage cutoff of 0.5, and maximum target number of 50. The BLASTp results were imported into MEGAN6 (Ultimate Edition, version 6.6) [48] for functional profiling with output of SEED Subsystem, Interpro2GO, KEGG, and COG categories. Exported tables of functional profiles were integrated for comparison of genomes.

### CRISPR array and CRISPR associated protein identification

CRISPR sites were identified using CRISPR Recognition Tool (CRT, version 1.2) with minimum repeat length (-minRL) of 15 bp, maximum repeat length (-maxRL) of 100 bp, minimum spacer length (-minSL) of 15 bp, maximum spacer length (-maxSL) of 100 bp, and repeat number (-minNR) of 5. The whole genome or draft genome sequences were annotated using PROKKA (*e* value 1e-10) and CRISPR associated proteins were counted for comparison. Phage/prophage proteins were identified from the functional gene classification of SEED at level 2.

### Phylogeny based on nearly full-length 16S rRNA from genomes/bins at phylum level

For the newly defined phyla in 2015 and 2016, 16S rRNA gene sequence and protein sequences of genome bins were downloaded under project PRJNA273161 [13] and PRJNA288027 [11] of NCBI. Protein sequences were used for completeness and contamination evaluation using CheckM (version) with the command lineage_wf. Only genome bins with completeness > 70%, contamination < 10%, and with 16S rRNA gene > 1300 bp were considered in this study. Genome bins with the highest completeness were selected as representatives of the phyla and their 16S rRNA genes (from genome annotation) were used for the phylogenetic tree construction. Genome bins of this study with completeness > 70%, contamination < 10%, and 16S rRNA gene > 1300 bp were included in the phylogenetic analysis. Representative 16S rRNA gene sequence of well-established bacterial and archaeal phyla from SILVA132 database were used as references. Sequences were aligned using MUSCLE (version 3.8.31) [38] with the default parameters, and the alignment were manually checked in MEGA7 (version 7.0.21) [49] to remove sequences introducing long gaps in the two ends. Gblocks (version 0.91bb) [50] was used to filter out poorly aligned positions with parameters of minimum length of a block 3 (-b4 = 3) and allowed gap positions of half (-b5 = h). The alignment was then converted into Phylip in format. RAxML (version 8.2.11) [51] was used to construct a maximum likelihood (ML) phylogenetic tree with parameters of substitution model GTRGAMMA and bootstrap analysis of 100 replicates (-f a -k -m GTRGAMMA -x 12345 -p 12345 -N 100). The ML tree in format of Newick and was then displayed in FigTree (version 1.4.3).

### Phylogeny based on concatenated rp16 ribosomal protein sequences

Representative genomes of well-established phyla and genome bins of *Patescibacteria* (from this study and recently defined CPR) were used for phylogenetic analysis. Sixteen single-copy ribosomal protein genes were used for phylogenetic inference. These include rpL2, rpL3, rpL4, rpL5, rpL6, rpL14, rpL15, rpL16, rpL18, rpL22, rpL24, rpS3, rpS8, rpS10, rpS17, and rpS19. PROKKA was used for identification of these proteins. MUSCLE was used for individual alignment of the genes with default parameters. Genes present in all species were concatenated using a customized Perl script and the alignment was then converted into Phylip in format. RAxML (version 8.2.11) [51] was used to construct a maximum likelihood phylogenetic tree with parameters of substitution model LG+GAMMA and bootstrap analysis of 100 replicates (-f a -k -m PROTGAMMALG -x 12345 -p 12345 -N 100). The ML tree in format of Newick and was then displayed in FigTree (version 1.4.3).

### Statistical analysis

The relative abundances of functional categories were expressed as mean ± standard error of mean (SEM). The differences of the functional genes and CRISPR spacers between the superphylum *Patescibacteria* and the other phyla were tested using Welch’s *T* test. The differences of abundance of the superphylum *Patescibacteria* in the 0.2-, 3-, and 10-μm filters were tested using Wilcoxon signed-rank test. The difference of abundance of the superphylum *Patescibacteria* in background (B), low (L), medium (M), and high (H) contamination wells were compared using Wilcoxon rank sum test.

## Supplementary information

**Additional file 1: Figure S1.** Heatmap showing the profile of contaminants in the background (B), low (L), mediate (M) and high (H) contamination wells. The concentrations of each contaminant were scaled linearly [(X-min)/max] to 0-1 for comparison. **Figure S2.** Maximum Likelihood (ML) tree of well-established bacterial and archaeal phyla, *Patescibacteria* superphylum and Candidate phyla of this study based on concatenated rp16 genes. **Figure S3.** Completeness evaluation of genome bins based on CheckM analysis (A) and number of unique tRNA genes (B). **Figure S4.** Comparison of ribosomal protein genes in the *Patescibacteria* superphylum and the other phyla. **Figure S5.** The rarefaction curve of the sequences showing the diversity index of observed OTUs and Shannon. The curves were based on calculation of diversity with step size of 2000 and iteration number of 10. **Figure S6** The enrichment of *Patescibacteria* cells on the 0.2-micron filter in comparison to the 10 or 3 micron filter (showing 108 sample with *Patescibacteria* abundance >0.5% in 0.2-micron filter). **Figure S7.** Comparison of transporter proteins in the superphylum *Patescibacteria* and other phyla. **Figure S8.** Number of CRISPR proteins (A) and CRISPR spacers (B) in the non-*Patescibacteria* phyla of Jillian Banfield’s binning. **Figure S9.** Number of CRISPR proteins (A) and CRISPR spacers (B) in the non-*Patescibacteria* phyla of this study. **Figure S10.** A heatmap of comparison of phage-associated proteins including phage structure, phage integration and regulation in the *Patescibacteria* superphylum and the other phyla. The color indicates the standardized number of phage proteins in each genome / bin.**Table S1.** Metadata of the ground water samples. See Figure S1 for the contaminant concentrations and the categorization of wells. **Table S2.** Summary of the raw reads and qualified reads of the metagenomic data. **Table S3.** Metagenomic assembly of the samples plus supplementary sequence. Contigs with length >500 bp were summarized. * The assembly of the sample FW301-10 failed due to super large data size and high diversity of community. **Table S4.** The alignment rates of each sample’s reads to its assembly. The alignment rates were calculated by Bowtie2 and Samtools. **Table S5.** Profiles of genome bins generated by MetaBat. Only genome bins with completeness >70% and contamination <10% are listed. **Table S6.** Genome sizes of *Patescibacteria* and non-*Patescibacteria* members including well-established phyla, newly defined phyla and genome bins of this study.

## Data Availability

The 16S rRNA gene amplicon sequences were submitted to NCBI database SRA under the project PRJNA514085 with accession SRR8427255. The shotgun metagenomic sequences were submitted to NCBI database SRA under the project PRJNA513876 with accession SRR8426587 - SRR8426598. The genomic sequences of genome bins from the genome-resolved metagenomic analysis were submitted to NCBI database WGS under the project PRJNA514088.
