# Supplementary material for: Small and mighty: adaptation of superphylum Patescibacteria to groundwater environment drives their genome simplicity
Source: Microbiome. 2020 Apr 6;8:51. doi: 10.1186/s40168-020-00825-w (PMC7137472; doi:10.1186/s40168-020-00825-w)
Supplement: Supplementary file 2 — Additional file 1: Figure S1. Heatmap showing the profile of contaminants in the background (B), low (L), mediate (M) and high (H) contamination wells. The concentrations of each contaminant were scaled linearly [(X-min)/max] to 0-1 for comparison. Figure S2. Maximum Likelihood (ML) tree of well-established bacterial and archaeal phyla, Patescibacteria superphylum and Candidate phyla of this study based on concatenated rp16 genes. Figure S3. Completeness evaluation of genome bins based on CheckM analysis (A) and number of unique tRNA genes (B). Figure S4. Comparison of ribosomal protein genes in the Patescibacteria superphylum and the other phyla. Figure S5. The rarefaction curve of the sequences showing the diversity index of observed OTUs and Shannon. The curves were based on calculation of diversity with step size of 2000 and iteration number of 10. Figure S6 The enrichment of Patescibacteria cells on the 0.2-micron filter in comparison to the 10 or 3 micron filter (showing 108 sample with Patescibacteria abundance >0.5% in 0.2-micron filter). Figure S7. Comparison of transporter proteins in the superphylum Patescibacteria and other phyla. Figure S8. Number of CRISPR proteins (A) and CRISPR spacers (B) in the non-Patescibacteria phyla of Jillian Banfield’s binning. Figure S9. Number of CRISPR proteins (A) and CRISPR spacers (B) in the non-Patescibacteria phyla of this study. Figure S10. A heatmap of comparison of phage-associated proteins including phage structure, phage integration and regulation in the Patescibacteria superphylum and the other phyla. The color indicates the standardized number of phage proteins in each genome / bin.Table S1. Metadata of the ground water samples. See Figure S1 for the contaminant concentrations and the categorization of wells. Table S2. Summary of the raw reads and qualified reads of the metagenomic data. Table S3. Metagenomic assembly of the samples plus supplementary sequence. Contigs with length >500 bp were summarized. * The [file 40168_2020_825_MOESM1_ESM.docx]

**Supplementary materials**


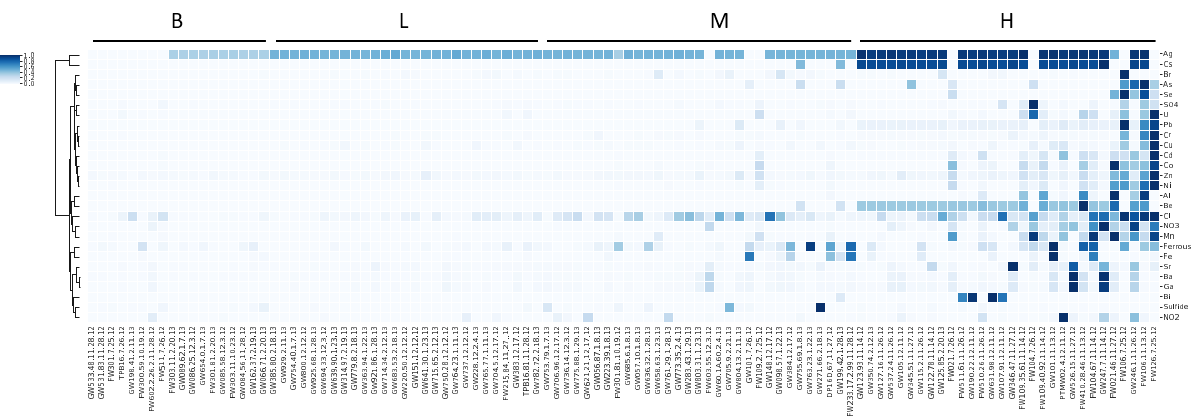
**Figure S1.** Heatmap showing the profile of contaminants in the background (B), low (L), mediate (M) and high (H) contamination wells. The concentrations of each contaminant were scaled linearly [(X-min)/max] to 0-1 for comparison.


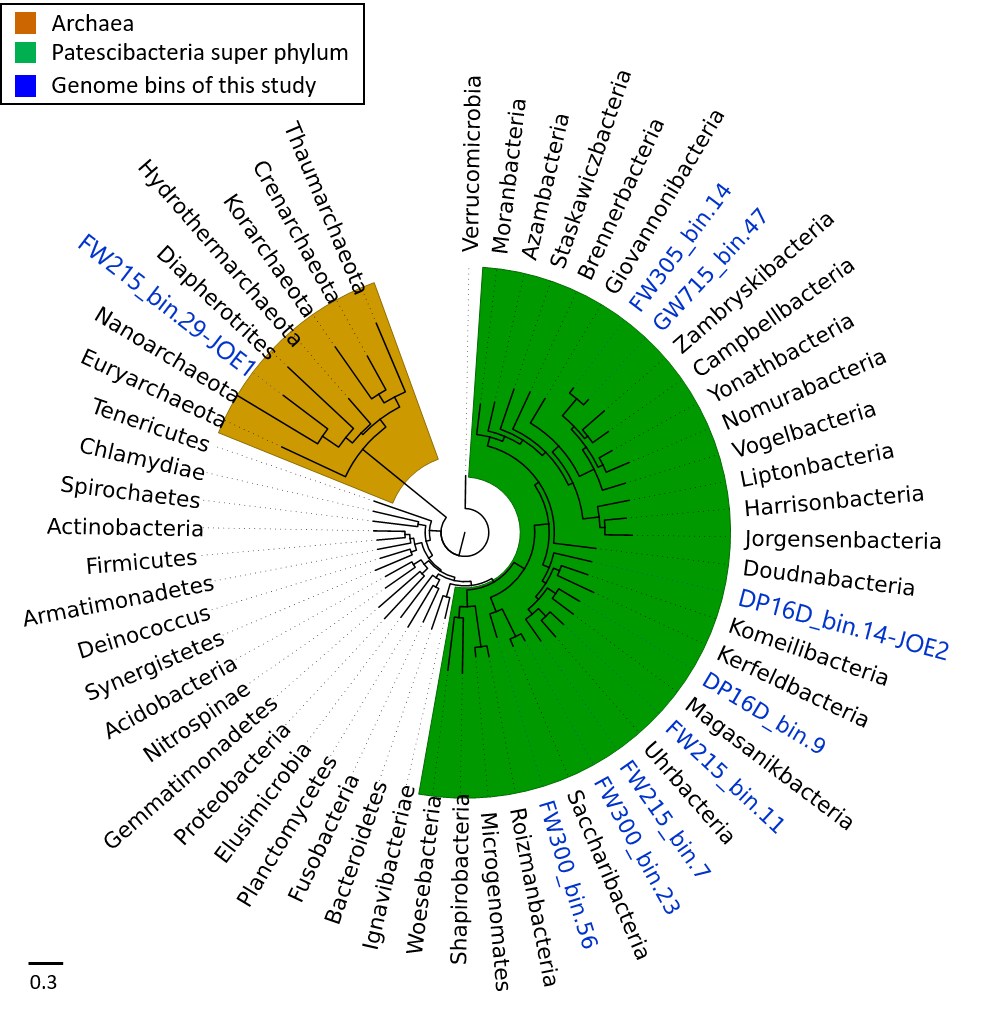


**Figure S2.** Maximum Likelihood (ML) tree of well-established bacterial and archaeal phyla, *Patescibacteria* superphylum and Candidate phyla of this study based on concatenated rp16 genes.


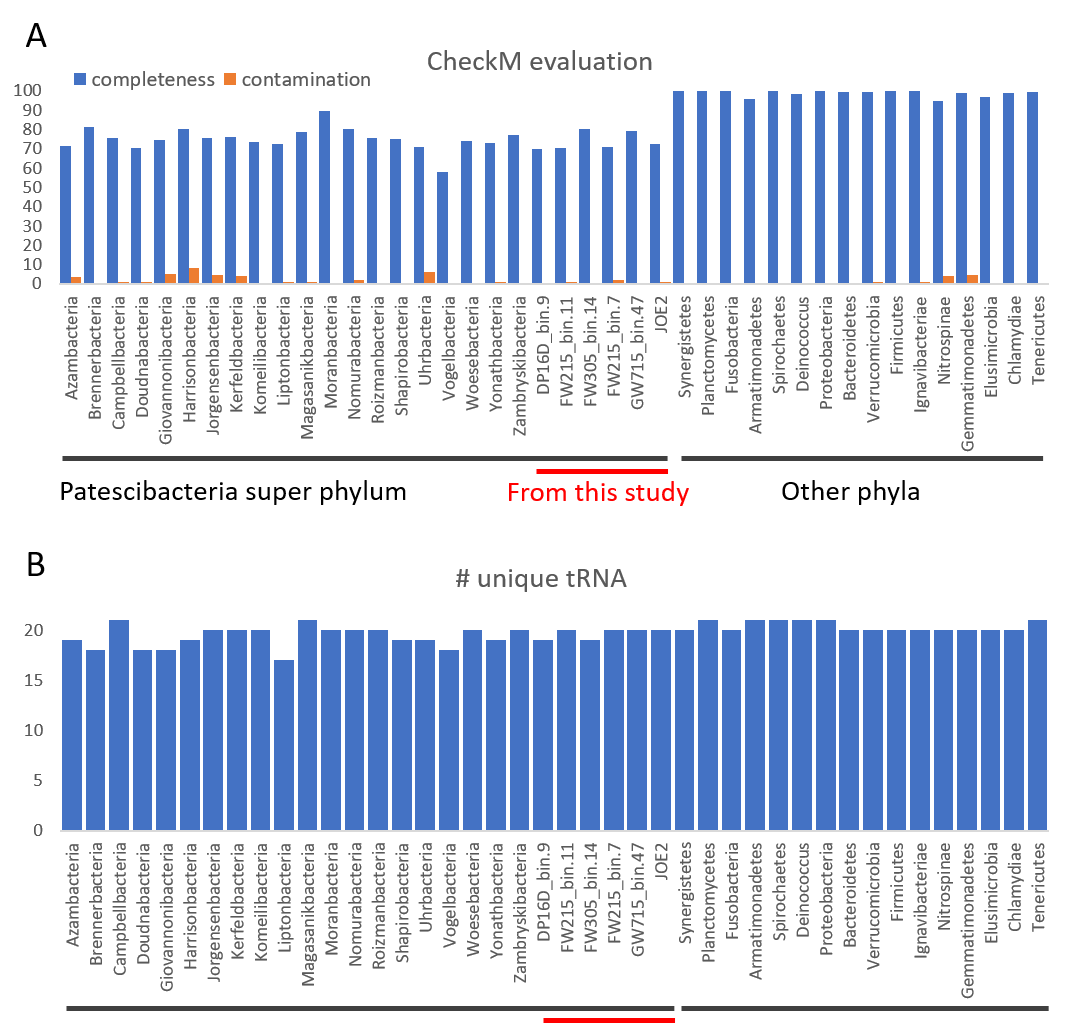


**Figure S3.** Completeness evaluation of genome bins based on CheckM analysis (A) and number of unique tRNA genes (B).


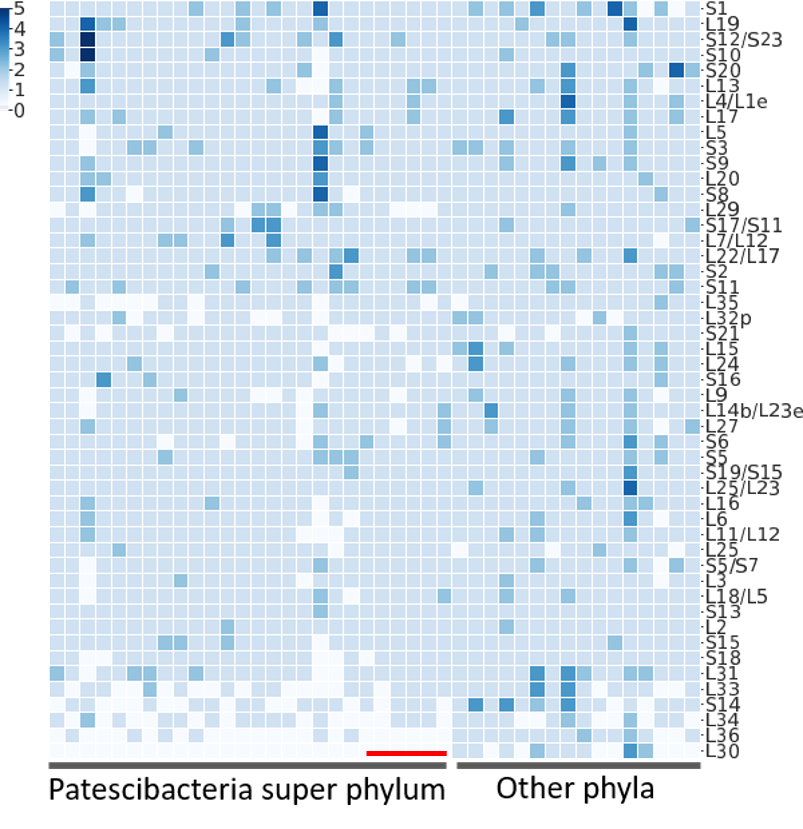


**Figure S4.** Comparison of ribosomal protein genes in the *Patescibacteria* superphylum and the other phyla.


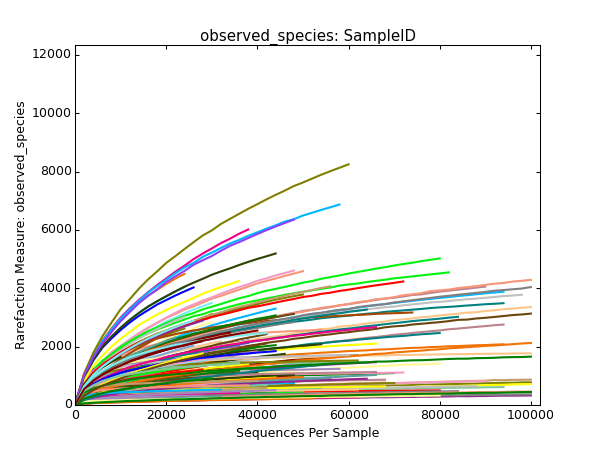


**Figure S5.** The rarefaction curve of the sequences showing the diversity index of observed OTUs and Shannon. The curves were based on calculation of diversity with step size of 2000 and iteration number of 10.


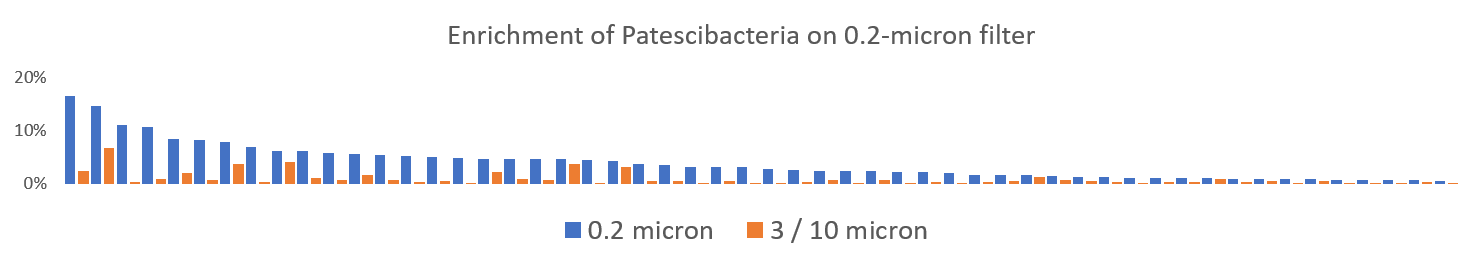


**Figure S6** The enrichment of *Patescibacteria* cells on the 0.2-micron filter in comparison to the 10 or 3 micron filter (showing 108 sample with *Patescibacteria* abundance >0.5% in 0.2-micron filter).


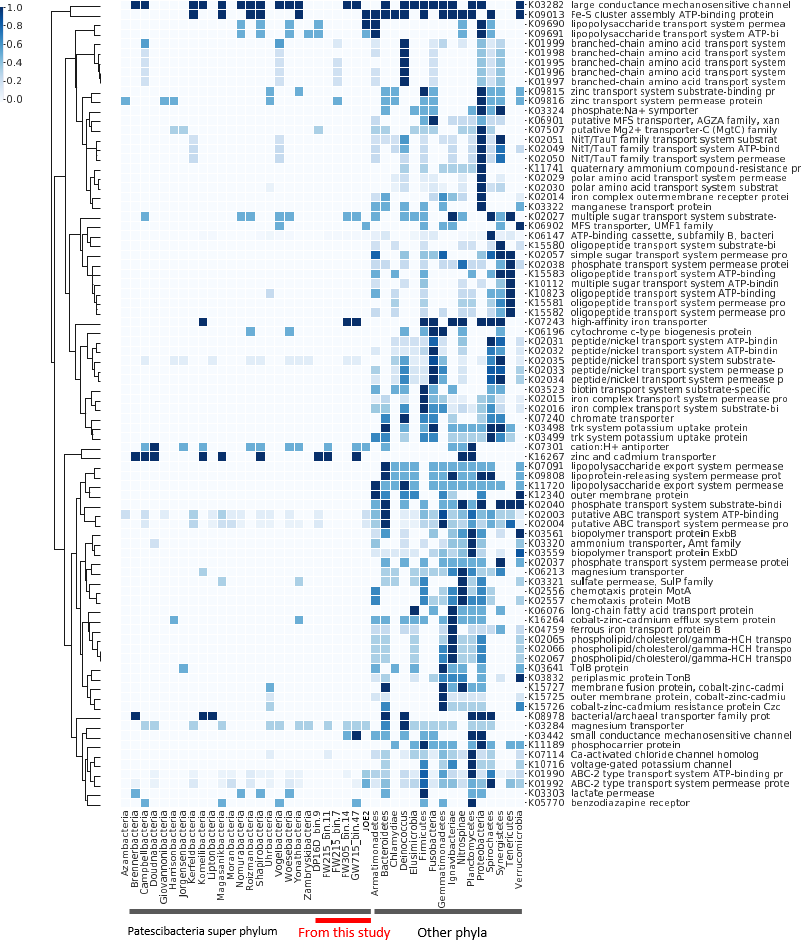


**Figure S7.** Comparison of transporter proteins in the superphylum *Patescibacteria* and other phyla.


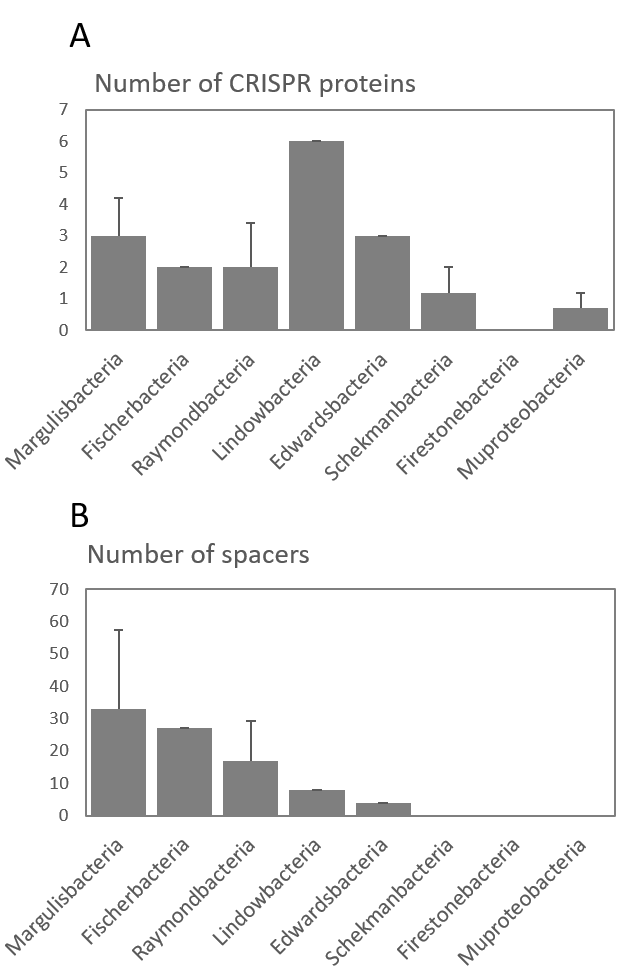


**Figure S8.** Number of CRISPR proteins (A) and CRISPR spacers (B) in the non-*Patescibacteria* phyla of Jillian Banfield’s binning.


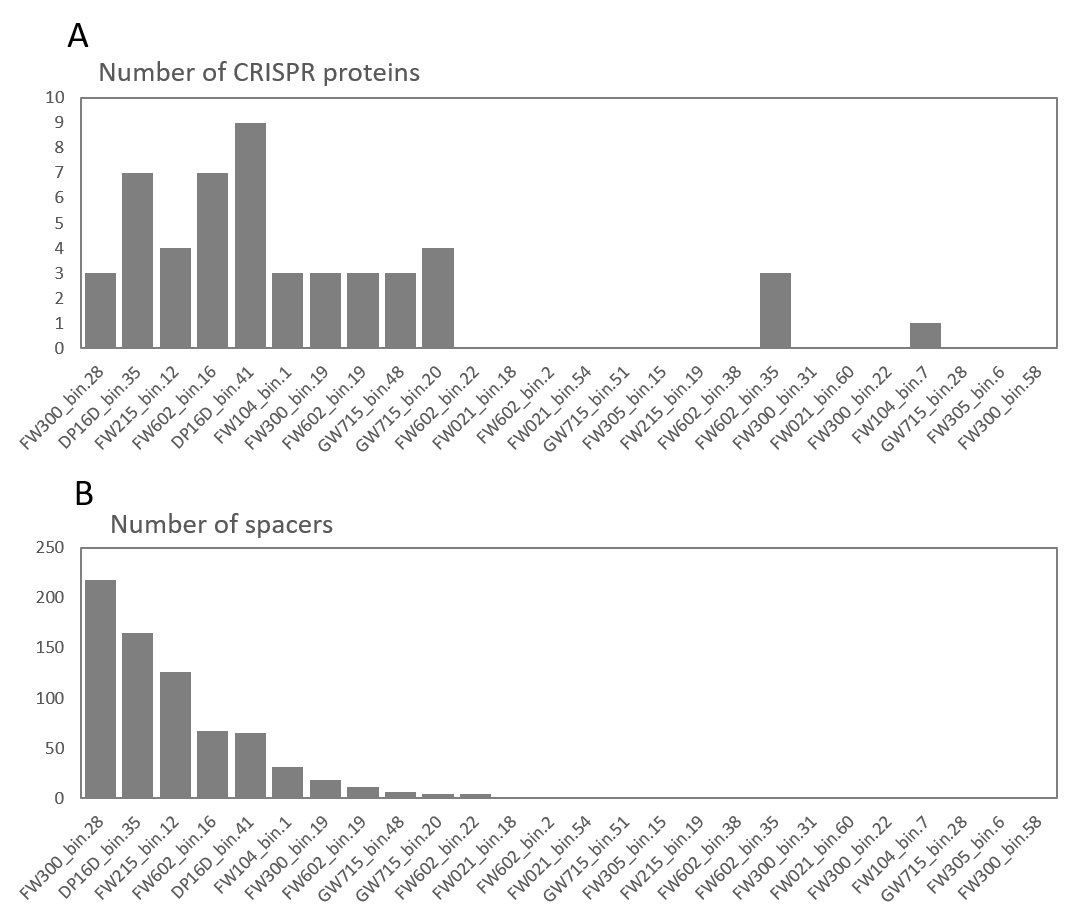


**Figure S9.** Number of CRISPR proteins (A) and CRISPR spacers (B) in the non-*Patescibacteria* phyla of this study.


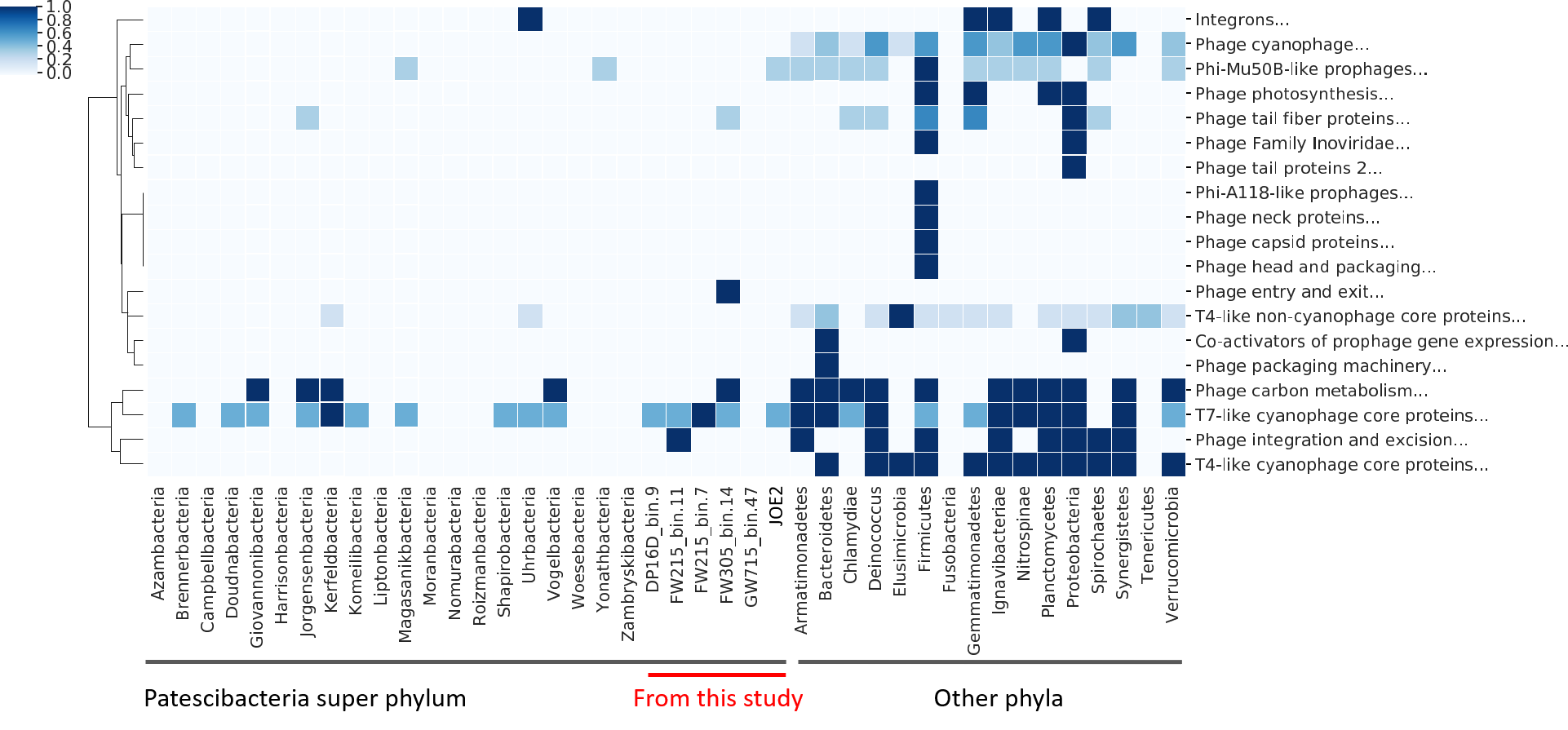
**Figure S10.** A heatmap of comparison of phage-associated proteins including phage structure, phage integration and regulation in the *Patescibacteria* superphylum and the other phyla. The color indicates the standardized number of phage proteins in each genome / bin.

**Table S1.** Metadata of the ground water samples. See Figure S1 for the contaminant concentrations and the categorization of wells.

| Sample ID | Collection Date | Longitude | Latitude | Depth (m) | Conductivity (S/m) | Dissovled Oxygen (mg/L) | Temperature (°C) | pH |
| --- | --- | --- | --- | --- | --- | --- | --- | --- |
| DP16D-67-11-27-12 | 11/27/2012 | -84.274678 | 35.9755 | 22.2 | 661 | 0.26 | 17.05 | 6.67 |
| FW021-46-11-27-12 | 11/27/2012 | -84.272033 | 35.977747 | 22.0 | 7967 | 0.27 | 16.41 | 3.43 |
| FW104-67-11-14-12 | 11/14/2012 | -84.273562 | 35.97736 | 44.4 | 13660 | 1.18 | 14.57 | 5.22 |
| FW106-11-13-12 | 11/13/2012 | -84.273484 | 35.977298 | 45.0 | 7864 | 0.18 | 14.84 | 3.55 |
| FW109-35-61-11-14-12 | 11/14/2012 | -84.273577 | 35.977374 | 34.9 | 1531 | 0.19 | 16.89 | 3.68 |
| FW109-40-92-11-14-12 | 11/14/2012 | -84.273577 | 35.977374 | 39.8 | 2726 | 0.29 | 12.42 | 4.08 |
| FW126-7-25-12 | 7/25/2012 | -84.273414 | 35.977358 | 49.6 | 16780 | 0.2 | 33.95 | 3.1 |
| FW215-84-11-27--12 | 11/27/2012 | -84.274722 | 35.975715 | 18.6 | 637.4 | 0.13 | 17.88 | 6.6 |
| FW233-17-2-99-11-28-12 | 11/28/2012 | -84.274723 | 35.975743 | 17.3 | 801.9 | 0.26 | 15.74 | 6.37 |
| FW300-59-10-19-12 | 10/19/2012 | -84.336176 | 35.941092 | 25.0 | 378.9 | 0.28 | 15.54 | 6.59 |
| FW301-7-25-12 | 7/25/2012 | -84.336181 | 35.941069 | 21.9 | 205 | 0.45 | 22.6 | 6.08 |
| FW301-81-10-19-12 | 10/19/2012 | -84.336181 | 35.941069 | 21.9 | 334.4 | 0.78 | 15.77 | 6.68 |
| FW301-81-2-20-13 | 2/20/2013 | -84.336181 | 35.941069 | 21.9 | 44.19 | 3.58 | 12.2 | 5.42 |
| FW303-11-10-23-12 | 10/23/2012 | -84.336279 | 35.941139 | 20.2 | 316.4 | 0.71 | 15.82 | 7.16 |
| FW303-11-2-20-13 | 2/20/2013 | -84.336279 | 35.941139 | 20.2 | 304.5 | 1.75 | 12.86 | 7.28 |
| FW410-28-46-11-13-12 | 11/13/2012 | -84.273957 | 35.977136 | 27.5 | 3355 | 0.34 | 16.02 | 3.69 |
| FW510-26-11-26-12 | 11/26/2012 | -84.272082 | 35.978585 | 25.8 | 1800 | 0.14 | 16.37 | 4.01 |
| FW511-61-11-26-12 | 11/26/2012 | -84.272035 | 35.978501 | 22.3 | 308.9 | 5.97 | 18.95 | 4.96 |
| FW511-7-26-12 | 7/26/2012 | -84.272035 | 35.978501 | 22.3 | 297.4 | 1.32 | 23.23 | 5.01 |
| FW602-2-26-2-11-28-12 | 11/28/2012 | -84.278931 | 35.974046 | 25.9 | 1704 | 0.41 | 16.42 | 6.48 |
| FW603-55-12-3-12 | 12/3/2012 | -84.278791 | 35.973802 | 32.8 | 6913 | 0.54 | 17.85 | 6.08 |
| GW056-87-1-8-13 | 1/8/2013 | -84.300532 | 35.957762 | 18.1 | 784.1 | 0.66 | 12.87 | 6.87 |
| GW057-10-1-8-13 | 1/8/2013 | -84.300523 | 35.957737 | 55.2 | 1003 | 0.71 | 15.08 | 6.72 |
| GW066-71-2-20-13 | 2/20/2013 | -84.281617 | 35.9708 | 22.8 | 69.32 | 0.48 | 11.91 | 6.2 |
| GW084-56-11-28-12 | 11/28/2012 | -84.28314 | 35.973088 | 27.8 | 339.7 | 0.19 | 15.66 | 7.15 |
| GW085-58-12-3-12 | 12/3/2012 | -84.281474 | 35.9725 | 58.8 | 365.1 | 2.5 | 19.2 | 7.29 |
| GW086-25-12-3-12 | 12/3/2012 | -84.281497 | 35.972458 | 29.6 | 275.1 | 0.63 | 20.36 | 6.47 |
| GW089-62-1-7-13 | 1/7/2013 | -84.299049 | 35.965917 | 25.0 | 327.8 | 0.37 | 12.94 | 7.95 |
| GW098-57-1-22-13 | 1/22/2013 | -84.286314 | 35.968022 | 103.4 | 1331 | 0.11 | 16.01 | 6.43 |
| GW101-11-13-12 | 11/13/2012 | -84.274117 | 35.97732 | 16.3 | 1721 | 0.96 | 18.12 | 6.81 |
| GW101-7-26-12 | 7/26/2012 | -84.274117 | 35.97732 | 16.3 | 934.8 | 1.06 | 24.98 | 7.2 |
| GW105-12-11-12 | 12/11/2012 | -84.271679 | 35.979239 | 16.1 | 1674 | 0.39 | 14.72 | 6.43 |
| GW107-91-12-11-12 | 12/11/2012 | -84.269997 | 35.979044 | 12.5 | 2819 | 0.37 | 20.36 | 6.18 |
| GW115-2-11-26-12 | 11/26/2012 | -84.273333 | 35.980507 | 52.0 | 581.6 | 0.41 | 17.45 | 7.03 |
| GW122-78-11-14-12 | 11/14/2012 | -84.273278 | 35.976124 | 142.0 | 1454 | 0 | 16.66 | 6.64 |
| GW123-93-11-14-12 | 11/14/2012 | -84.273314 | 35.976106 | 572.0 | 1631 | 0.02 | 16.61 | 9.99 |
| GW125-85-2-20-13 | 2/20/2013 | -84.271975 | 35.976523 | 552.0 | 2538 | 0.51 | 14.19 | 9.99 |
| GW127-16-11-26-12 | 11/26/2012 | -84.273424 | 35.976405 | 22.8 | 824.9 | 0.12 | 16.97 | 6.55 |
| GW148-12-17-12 | 12/17/2012 | -84.238597 | 35.993332 | 10.6 | 1399 | 1.32 | 16.53 | 6.45 |
| GW151-12-12-12 | 12/12/2012 | -84.236973 | 35.993413 | 96.0 | 432.2 | 0.25 | 15.4 | 7.07 |
| GW162-37-2-19-13 | 2/19/2013 | -84.296806 | 35.968495 | 125.0 | 247.6 | 0.26 | 14.3 | 8.21 |
| GW190-22-12-11-12 | 12/11/2012 | -84.271177 | 35.980705 | 25.9 | 345.4 | 0.92 | 17.91 | 6.69 |
| GW198-41-2-11-13 | 2/11/2013 | -84.244751 | 35.994113 | 26.5 | 468.2 | 3.48 | 16.1 | 6.06 |
| GW199-42-1-28-13 | 1/28/2013 | -84.244432 | 35.993718 | 22.5 | 581.6 | 0.5 | 14.29 | 6.53 |
| GW220-50-12-12-12 | 12/12/2012 | -84.236976 | 35.993381 | 44.7 | 410.7 | 0.52 | 13.09 | 7.49 |
| GW223-39-1-8-13 | 1/8/2013 | -84.239517 | 35.991954 | 90.0 | 466.5 | 0.41 | 16.41 | 6.83 |
| GW228-12-2-4-13 | 2/4/2013 | -84.283453 | 35.96866 | 100.0 | 282.4 | 0.17 | 10.92 | 9.31 |
| GW246-1-11-26-12 | 11/26/2012 | -84.272935 | 35.977144 | 74.6 | 18580 | 0.19 | 17.56 | 4.48 |
| GW247-7-11-14-12 | 11/14/2012 | -84.272726 | 35.9773 | 74.9 | 20620 | 0.09 | 17.5 | 5.88 |
| GW271-66-2-18-13 | 2/18/2013 | -84.27058705 | 35.97989558 | 29.4 | 521.9 | 0.03 | 15.57 | 7.71 |
| GW283-43-1-29-13 | 1/29/2013 | -84.244414 | 35.991366 | 20.0 | 1089 | 0.07 | 18.01 | 6.95 |
| GW314-97-2-19-13 | 2/19/2013 | -84.27177607 | 35.97587976 | 113.0 | 602.7 | 0.23 | 14.69 | 7.37 |
| GW345-51-11-27-12 | 11/27/2012 | -84.276925 | 35.97508 | 26.0 | 183.8 | 1.19 | 15.07 | 5.23 |
| GW346-47-11-27-12 | 11/27/2012 | -84.276916 | 35.975088 | 64.6 | 6931 | 0.26 | 14.79 | 6.58 |
| GW350-74-2-12-13 | 2/12/2013 | -84.268309 | 35.978922 | 43.4 | 545 | 0.02 | 17.98 | 6.67 |
| GW363-94-1-22-13 | 1/22/2013 | -84.287521 | 35.969048 | 75.0 | 424.9 | 0.03 | 15.28 | 9.13 |
| GW383-12-17-12 | 12/17/2012 | -84.239422 | 35.992878 | 23.1 | 521.7 | 4.54 | 17.43 | 7.11 |
| GW384-12-17-12 | 12/17/2012 | -84.239429 | 35.992922 | 55.7 | 802.9 | 0.08 | 16.95 | 6.64 |
| GW385-80-2-18-13 | 2/18/2013 | -84.239425 | 35.992899 | 178.7 | 780.09 | 0.29 | 15.36 | 9.19 |
| GW526-15-11-27-12 | 11/27/2012 | -84.276907 | 35.975102 | 123.0 | 9468 | 0.14 | 14.54 | 7.95 |
| GW531-83-11-28-12 | 11/28/2012 | -84.281558 | 35.973944 | 38.2 | 39.68 | 4.32 | 15.07 | 5.49 |
| GW533-48-11-28-12 | 11/28/2012 | -84.281393 | 35.974108 | 30.1 | 118 | 1.54 | 15.92 | 6.27 |
| GW537-24-11-26-12 | 11/26/2012 | -84.280229 | 35.973362 | 23.0 | 2618 | 0.05 | 15.6 | 6.49 |
| GW60-1A-60-2-4-13 | 2/4/2013 | -84.248782 | 35.987834 | 23.2 | 1076 | 0.23 | 19.23 | 7.38 |
| GW621-21-12-17-12 | 12/17/2012 | -84.2909 | 35.964088 | 40.5 | 356.4 | 0.17 | 14.64 | 7.19 |
| GW631-98-12-11-12 | 12/11/2012 | -84.2704 | 35.97923 | 15.0 | 318.8 | 0.26 | 16.86 | 4.32 |
| GW636-32-1-28-13 | 1/28/2013 | -84.287902 | 35.967252 | 117.0 | 317.9 | 0.2 | 12.17 | 8.87 |
| GW639-90-1-23-13 | 1/23/2013 | -84.291404 | 35.965809 | 125.5 | 845.3 | 0.32 | 12.05 | 9.2 |
| GW641-30-1-23-13 | 1/23/2013 | -84.291542 | 35.965931 | 24.0 | 25.59 | 7.22 | 12.64 | 5.25 |
| GW654-0-1-7-13 | 1/7/2013 | -84.302538 | 35.962761 | 15.3 | 269 | 2.35 | 13.01 | 7.19 |
| GW658-63-1-23-13 | 1/23/2013 | -84.244105 | 35.991761 | 18.8 | 454.3 | 0.03 | 19.06 | 6.4 |
| GW683-53-2-18-13 | 2/18/2013 | -84.299249 | 35.957053 | 196.8 | 393.6 | 5.42 | 13.82 | 7.49 |
| GW685-6-1-8-13 | 1/8/2013 | -84.300271 | 35.957771 | 138.3 | 906.1 | 0.1 | 14.55 | 6.88 |
| GW694-33-12-3-12 | 12/3/2012 | -84.290954 | 35.963467 | 204.5 | 444.6 | 0.15 | 15.11 | 7.3 |
| GW704-5-12-17-12 | 12/12/2012 | -84.290837 | 35.96353 | 256.0 | 529.2 | 0.99 | 13.7 | 7.62 |
| GW705-9-2-21-13 | 2/21/2013 | -84.29107919 | 35.96372889 | 307.0 | 891.7 | 0.33 | 12.37 | 9.19 |
| GW706-96-12-17-12 | 12/17/2012 | -84.291005 | 35.963775 | 182.5 | 710.4 | 0.04 | 14.11 | 7.88 |
| GW714-34-2-12-13 | 2/12/2013 | -84.313845 | 35.949511 | 145.0 | 514.2 | 0.14 | 13.92 | 7.48 |
| GW715-65-2-12-13 | 2/12/2013 | -84.313801 | 35.949545 | 43.1 | 532.9 | 5.47 | 14.59 | 6.65 |
| GW736-14-12-3-12 | 12/3/2012 | -84.280642 | 35.970896 | 102.4 | 916.7 | 0.13 | 15.88 | 6.64 |
| GW737-12-12-12 | 12/12/2012 | -84.280741 | 35.97079 | 89.4 | 937.2 | 0.33 | 13.53 | 6.66 |
| GW750-28-12-12-12 | 12/12/2012 | -84.235312 | 35.994376 | 72.3 | 367.6 | 0.23 | 15.12 | 7.11 |
| GW753-79-1-7-13 | 1/7/2012 | -84.241126 | 35.993391 | 70.3 | 215 | 0.17 | 15.94 | 10.48 |
| GW754-40-1-7-13 | 1/7/2013 | -84.241189 | 35.993378 | 23.9 | 176.6 | 1.29 | 15.91 | 3.13 |
| GW755-69-1-8-13 | 1/8/2013 | -84.242031 | 35.994769 | 59.9 | 386.4 | 1.12 | 14.9 | 9.34 |
| GW761-29-1-28-13 | 1/28/2013 | -84.250491 | 35.989904 | 15.2 | 380.9 | 4.72 | 15.96 | 6.59 |
| GW763-23-1-22-13 | 1/22/2013 | -84.240111 | 35.992222 | 64.8 | 699.5 | 0.13 | 17.11 | 6.65 |
| GW764-23-1-11-13 | 1/11/2013 | -84.257055 | 35.989269 | 31.5 | 197.1 | 0.95 | 19.06 | 8.57 |
| GW765-77-1-11-13 | 1/11/2013 | -84.256997 | 35.989297 | 58.6 | 638.8 | 0.23 | 19.14 | 6.68 |
| GW773-35-2-4-13 | 2/4/2013 | -84.246683 | 35.991147 | 56.2 | 330.1 | 0.24 | 13.43 | 7.46 |
| GW775-88-1-29-13 | 1/29/2013 | -84.245848 | 35.989595 | 58.0 | 583.2 | 0.97 | 19.12 | 7.2 |
| GW779-8-2-18-13 | 2/18/2013 | -84.25334197 | 35.9886503 | 62.0 | 460.9 | 0.2 | 16.74 | 9.55 |
| GW782-72-2-18-13 | 2/18/2013 | -84.255601 | 35.985734 | 34.9 | 556.5 | 2.65 | 17.49 | 7.52 |
| GW800-12-12-12 | 12/12/2012 | -84.28178 | 35.968949 | 29.4 | 379.1 | 8.01 | 13.13 | 6.7 |
| GW803-31-1-23-13 | 1/23/2013 | -84.243755 | 35.991689 | 23.2 | 397.7 | 5.11 | 13.68 | 5.4 |
| GW804-13-2-11-13 | 2/11/2013 | -84.24405271 | 35.99152444 | 23.5 | 603.1 | 6.57 | 15.48 | 4.91 |
| GW921-86-1-28-13 | 1/28/2013 | -84.287507 | 35.970344 | 48.0 | 465.5 | 0.64 | 15.57 | 7.46 |
| GW925-68-1-28-13 | 1/28/2013 | -84.287535 | 35.970325 | 147.0 | 654.7 | 0.35 | 11.97 | 9.79 |
| GW928-54-2-11-13 | 2/11/2013 | -84.264657 | 35.987421 | 43.2 | 241.5 | 0.21 | 16.65 | 6.69 |
| GW929-2-11-13 | 2/11/2013 | -84.264601 | 35.987452 | 28.0 | 34.79 | 3.09 | 16.07 | 5.18 |
| PTMW02-4-12-11-12 | 12/11/2012 | -84.276332 | 35.975573 | 44.7 | 9307 | 0.16 | 14.42 | 4.72 |
| TPB16-7-26-12 | 7/26/2012 | -84.274687 | 35.975643 | 15.3 | 1444 | 0.18 | 26.79 | 7.1 |
| TPB16-81-11-28-12 | 11/28/2012 | -84.274687 | 35.975643 | 15.3 | 843.3 | 0.35 | 18.59 | 6.58 |

**Table S2.** Summary of the raw reads and qualified reads of the metagenomic data.

| Sample ID | Raw reads (million) | Raw base (Gbp) | Duplication | | Quality trimming and filtering | | | | | | | | Total base (HQ reads, Gbp) |
| --- | --- | --- | --- | --- | --- | --- | --- | --- | --- | --- | --- | --- | --- |
|  |  |  |  |  | Forward read | | | | Reverse read | | | |  |
|  |  |  | Deduplicated reads | Duplication rate | No. of HQ reads (million) | No. of bases (Gbp) | No. of HQ bases (Gbp) | Q20 (percentage of HQ bases) | No. of HQ reads (million) | No. of bases (Gbp) | No. of HQ bases (Gbp) | Q20 (percentage of HQ base) |  |
| DP16D | 218.2 | 32.9 | 213.7 | 2.1% | 99.9 | 15.0 | 14.6 | 97.2% | 99.9 | 15.0 | 14.0 | 93.8% | 30.0 |
| FW021 | 236.4 | 35.7 | 229.6 | 2.9% | 92.1 | 13.9 | 13.5 | 97.4% | 92.1 | 13.8 | 12.7 | 92.3% | 27.7 |
| FW104 | 237.7 | 35.9 | 168.7 | 29.0% | 50.1 | 7.5 | 7.1 | 95.1% | 50.1 | 7.5 | 6.5 | 87.5% | 15.0 |
| FW106 | 180.0 | 27.2 | 159.1 | 11.6% | 62.1 | 9.4 | 8.8 | 94.0% | 62.1 | 9.3 | 8.4 | 90.2% | 18.7 |
| FW106-02 | 300.6 | 45.4 | 289.1 | 3.8% | 133.6 | 20.1 | 19.4 | 96.7% | 133.6 | 20.0 | 18.7 | 93.3% | 40.1 |
| FW106-10 | 549.5 | 83.0 | 531.2 | 3.3% | 242.3 | 36.5 | 34.7 | 95.0% | 242.3 | 36.3 | 33.7 | 92.8% | 72.8 |
| FW215 | 190.5 | 28.8 | 186.8 | 2.0% | 87.6 | 13.2 | 12.8 | 97.2% | 87.6 | 13.1 | 12.2 | 93.2% | 26.3 |
| FW300 | 310.1 | 46.8 | 302.5 | 2.4% | 137.4 | 20.7 | 20.2 | 97.6% | 137.4 | 20.5 | 18.8 | 91.3% | 41.2 |
| FW301 | 190.7 | 28.8 | 187.3 | 1.8% | 84.3 | 12.7 | 12.4 | 97.5% | 84.3 | 12.6 | 11.7 | 92.5% | 25.3 |
| FW301-02 | 373.1 | 56.3 | 345.7 | 7.4% | 169.3 | 25.5 | 25.1 | 98.4% | 169.3 | 25.5 | 24.6 | 96.7% | 51.0 |
| FW301-10 | 341.8 | 51.6 | 315.1 | 7.8% | 154.3 | 23.3 | 22.9 | 98.5% | 154.3 | 23.2 | 22.5 | 96.8% | 46.5 |
| FW305 | 178.9 | 27.0 | 135.6 | 24.2% | 50.5 | 7.6 | 7.4 | 96.9% | 50.5 | 7.6 | 6.8 | 90.3% | 15.2 |
| FW602 | 174.1 | 26.3 | 170.4 | 2.1% | 79.1 | 11.9 | 11.6 | 97.1% | 79.1 | 11.8 | 11.0 | 93.1% | 23.7 |
| GW199 | 229.0 | 34.6 | 124.3 | 45.7% | 41.7 | 6.3 | 6.0 | 95.3% | 41.7 | 6.2 | 5.5 | 89.0% | 12.5 |
| GW715 | 264.1 | 39.9 | 244.2 | 7.5% | 104.3 | 15.7 | 15.3 | 97.6% | 104.3 | 15.6 | 14.3 | 91.8% | 31.3 |
| GW928 | 239.2 | 36.1 | 195.9 | 18.1% | 49.0 | 7.4 | 7.0 | 95.6% | 49.0 | 7.3 | 6.5 | 89.0% | 14.7 |
| Total | 4213.7 | 636.3 | 3799.1 | - | 1637.8 | 246.5 | 238.8 | - | 1637.8 | 245.4 | 228.1 | - | 491.8 |
| Mean | 263.4 | 39.8 | 237.4 | 10.7% | 102.4 | 15.4 | 14.9 | 96.7% | 102.4 | 15.3 | 14.3 | 92.1% | 30.7 |
| SD | 97.1 | 14.7 | 102.3 | 12.5% | 54.0 | 8.1 | 7.9 | 1.3% | 54.0 | 8.1 | 7.8 | 2.6% | 16.2 |

**Table S3.** Metagenomic assembly of the samples plus supplementary sequence. Contigs with length >500 bp were summarized.

| Sample ID | Contig number | Total base of contigs | Max sequence length | N50 length | GC content |
| --- | --- | --- | --- | --- | --- |
| DP16D | 118677 | 1.91E+08 | 273343 | 1952 | 54.77% |
| FW021 | 70823 | 2.09E+08 | 588991 | 8788 | 61.52% |
| FW104 | 13504 | 24809230 | 116275 | 2560 | 57.04% |
| FW106 | 18736 | 44868411 | 560079 | 12032 | 61.69% |
| FW106-02 | 18472 | 57771662 | 430515 | 11148 | 61.89% |
| FW106-10 | 43324 | 1.13E+08 | 430652 | 8514 | 60.03% |
| FW215 | 139593 | 2.01E+08 | 643366 | 1568 | 52.14% |
| FW300 | 175376 | 2.46E+08 | 735234 | 1453 | 55.77% |
| FW301 | 66427 | 86231473 | 199693 | 1279 | 60.14% |
| FW301-02 | 91051 | 1.18E+08 | 251934 | 1273 | 55.55% |
| FW305 | 42729 | 63729503 | 604198 | 1678 | 63.14% |
| FW602 | 133699 | 2.54E+08 | 1783802 | 2652 | 62.62% |
| GW199 | 6604 | 8090154 | 23002 | 1231 | 50.54% |
| GW715 | 135463 | 2.52E+08 | 1047723 | 2740 | 62.33% |
| GW928 | 45056 | 71729291 | 577390 | 1999 | 60.57% |

* The assembly of the sample FW301-10 failed due to super large data size and high diversity of community.

**Table S4.** The alignment rates of each sample’s reads to its assembly. The alignment rates were calculated by Bowtie2 and Samtools.

| Sample ID | # raw reads (million) | # base of raw reads (Gbp) | # base after deduplication, quality trimming and filtering (Gbp) | Percent of high quality reads mapping to assembly |
| --- | --- | --- | --- | --- |
| DP16D | 218.2 | 32.9 | 30.0 | 19.64% |
| FW021 | 236.4 | 35.7 | 27.7 | 73.13% |
| FW104 | 237.7 | 35.9 | 15.0 | 36.62% |
| FW106 | 180.0 | 27.2 | 18.7 | 73.09% |
| FW106-02 | 300.6 | 45.4 | 40.1 | 65.77% |
| FW106-10 | 549.5 | 83.0 | 72.8 | 63.16% |
| FW215 | 190.5 | 28.8 | 26.3 | 17.36% |
| FW300 | 310.1 | 46.8 | 41.2 | 8.47% |
| FW301 | 190.7 | 28.8 | 25.3 | 3.94% |
| FW301-02 | 373.1 | 56.3 | 51.0 | 3.35% |
| FW305 | 178.9 | 27.0 | 15.2 | 16.91% |
| FW602 | 174.1 | 26.3 | 23.7 | 30.72% |
| GW199 | 229.0 | 34.6 | 12.5 | 11.92% |
| GW715 | 264.1 | 39.9 | 31.3 | 23.74% |
| GW928 | 239.2 | 36.1 | 14.7 | 31.87% |

**Table S5**. Profiles of genome bins generated by MetaBat. Only genome bins with completeness >70% and contamination <10% are listed.

| Bin ID | Taxonomic classification (by pplacer of CheckM) | Complet  eness | Contam  ination | Contig number | Total base of contigs | Max sequence length | N50 length |
| --- | --- | --- | --- | --- | --- | --- | --- |
| DP16D_bin.48 | k__Bacteria_(UID3187) | 100 | 5.45 | 120 | 3.23 | 125,024 | 41,819 |
| FW021_bin.18 | k__Bacteria_(UID1452) | 100 | 1.85 | 100 | 2.87 | 394,163 | 148,845 |
| FW106-10_bin.31 | k__Bacteria_(UID203) | 100 | 8.62 | 333 | 4.06 | 60,718 | 20,711 |
| FW021_bin.60 | g__Mycobacterium_(UID1816) | 99.94 | 0.58 | 75 | 6.14 | 384,008 | 140,583 |
| GW715_bin.37 | c__Alphaproteobacteria_(UID3422) | 99.89 | 2.73 | 43 | 5.77 | 515,386 | 266,291 |
| FW106_bin.13 | c__Betaproteobacteria_(UID3959) | 99.62 | 2.56 | 54 | 5.99 | 560,079 | 200,020 |
| FW301_bin.9 | o__Burkholderiales_(UID4000) | 99.53 | 0.35 | 626 | 4.65 | 38,806 | 10,565 |
| FW021_bin.57 | o__Actinomycetales_(UID1593) | 99.49 | 1.26 | 41 | 3.95 | 516,796 | 325,858 |
| FW106_bin.9 | o__Burkholderiales_(UID4001) | 99.49 | 0.33 | 336 | 4.02 | 123,784 | 17,717 |
| FW106-10_bin.28 | o__Burkholderiales_(UID4001) | 99.49 | 0.2 | 165 | 3.09 | 93,290 | 33,103 |
| FW305_bin.1 | o__Actinomycetales_(UID1590) | 99.41 | 0 | 13 | 2.68 | 604,198 | 341,920 |
| FW300_bin.58 | c__Betaproteobacteria_(UID3888) | 99.36 | 0.88 | 26 | 2.48 | 312,961 | 212,466 |
| FW305_bin.9 | c__Gammaproteobacteria_(UID4274) | 99.21 | 1.36 | 157 | 4.60 | 249,922 | 52,387 |
| FW300_bin.35 | c__Alphaproteobacteria_(UID3422) | 99.08 | 2.23 | 95 | 4.91 | 318,114 | 97,883 |
| GW715_bin.44 | o__Burkholderiales_(UID4000) | 98.81 | 0 | 91 | 8.98 | 634,046 | 178,790 |
| FW021_bin.31 | p__Bacteroidetes_(UID2591) | 98.77 | 2.37 | 301 | 4.52 | 114,523 | 25,681 |
| FW021_bin.74 | k__Bacteria_(UID1452) | 98.77 | 1.85 | 87 | 3.03 | 196,054 | 59,548 |
| FW602_bin.22 | k__Bacteria_(UID1452) | 98.68 | 0.99 | 38 | 4.03 | 346,778 | 186,054 |
| se_bin.6 | f__Xanthomonadaceae_(UID4214) | 98.64 | 1.51 | 389 | 3.66 | 63,506 | 15,930 |
| DP16D_bin.19 | c__Gammaproteobacteria_(UID4274) | 98.61 | 1.04 | 144 | 4.49 | 214,456 | 50,311 |
| FW021_bin.21 | c__Betaproteobacteria_(UID3959) | 98.58 | 1.85 | 86 | 3.41 | 397,732 | 111,098 |
| GW715_bin.21 | o__Sphingomonadales_(UID3310) | 98.46 | 4.91 | 295 | 4.27 | 204,652 | 32,436 |
| FW602_bin.15 | c__Betaproteobacteria_(UID3971) | 98.42 | 6.68 | 87 | 5.85 | 600,586 | 161,352 |
| GW715_bin.18 | c__Betaproteobacteria_(UID3959) | 98.41 | 4.65 | 343 | 3.11 | 85,893 | 25,625 |
| FW106_bin.8 | o__Burkholderiales_(UID4000) | 98.36 | 1.87 | 38 | 4.25 | 418,061 | 154,406 |
| DP16D_bin.35 | k__Bacteria_(UID3187) | 98.18 | 2.73 | 145 | 3.63 | 273,343 | 56,966 |
| GW715_bin.29 | c__Alphaproteobacteria_(UID3422) | 98.05 | 3 | 114 | 3.12 | 188,491 | 55,486 |
| FW602_bin.18 | o__Rhizobiales_(UID3642) | 98.02 | 0.79 | 27 | 3.12 | 790,923 | 373,877 |
| FW106-10_bin.30 | f__Xanthomonadaceae_(UID4214) | 97.99 | 1.12 | 49 | 3.38 | 365,303 | 124,146 |
| FW301-02_bin.23 | o__Rhodospirillales_(UID3754) | 97.99 | 1.45 | 353 | 2.05 | 27,717 | 7,584 |
| FW215_bin.12 | k__Bacteria_(UID2570) | 97.98 | 0 | 47 | 4.20 | 643,366 | 175,842 |
| GW715_bin.31 | o__Rhodospirillales_(UID3754) | 97.97 | 1.49 | 50 | 6.46 | 924,814 | 338,475 |
| FW021_bin.58 | f__Xanthomonadaceae_(UID4214) | 97.82 | 2.5 | 136 | 3.60 | 164,026 | 40,809 |
| FW106-02_bin.9 | f__Xanthomonadaceae_(UID4214) | 97.66 | 3.35 | 308 | 3.53 | 69,506 | 17,558 |
| FW106-02_bin.10 | f__Xanthomonadaceae_(UID4214) | 97.52 | 1.21 | 456 | 3.60 | 73,209 | 12,337 |
| FW300_bin.28 | k__Bacteria_(UID3187) | 97.48 | 3.36 | 45 | 2.88 | 201,346 | 99,979 |
| FW106-02_bin.3 | f__Xanthomonadaceae_(UID4214) | 97.47 | 2.67 | 203 | 3.81 | 235,300 | 41,120 |
| FW021_bin.48 | k__Bacteria_(UID1452) | 97.38 | 3.94 | 90 | 3.19 | 173,571 | 62,330 |
| FW021_bin.27 | o__Burkholderiales_(UID4000) | 97.35 | 1.03 | 122 | 3.14 | 153,140 | 39,867 |
| FW021_bin.29 | f__Xanthomonadaceae_(UID4214) | 97.31 | 0.73 | 76 | 3.60 | 168,646 | 65,296 |
| se_bin.5 | f__Xanthomonadaceae_(UID4214) | 97.29 | 3.21 | 97 | 3.89 | 230,714 | 76,916 |
| FW106-10_bin.33 | f__Xanthomonadaceae_(UID4214) | 97.24 | 3.56 | 139 | 3.08 | 235,696 | 50,340 |
| FW106-02_bin.7 | c__Gammaproteobacteria_(UID4202) | 97.21 | 2.83 | 442 | 3.07 | 34,202 | 8,847 |
| FW301-02_bin.25 | o__Burkholderiales_(UID4000) | 97 | 3.54 | 453 | 4.91 | 76,807 | 15,656 |
| FW602_bin.35 | c__Gammaproteobacteria_(UID4267) | 96.8 | 0.38 | 13 | 3.53 | 1,783,802 | 1,783,802 |
| FW300_bin.52 | k__Bacteria_(UID3187) | 96.76 | 3.92 | 250 | 2.67 | 98,754 | 26,967 |
| GW928_bin.16 | k__Bacteria_(UID3187) | 96.76 | 3.64 | 23 | 3.26 | 577,390 | 321,837 |
| FW106-10_bin.21 | c__Betaproteobacteria_(UID3959) | 96.74 | 2.84 | 61 | 3.24 | 430,652 | 262,923 |
| GW715_bin.16 | c__Alphaproteobacteria_(UID3305) | 96.61 | 0.65 | 66 | 6.02 | 363,860 | 176,897 |
| GW715_bin.19 | f__Rhodocyclaceae_(UID3972) | 96.56 | 1.04 | 151 | 2.67 | 137,154 | 36,883 |
| FW106_bin.6 | c__Gammaproteobacteria_(UID4202) | 96.49 | 1.36 | 139 | 3.78 | 241,417 | 55,622 |
| FW602_bin.10 | c__Betaproteobacteria_(UID3959) | 96.45 | 1.18 | 169 | 3.56 | 106,972 | 30,446 |
| GW715_bin.15 | c__Deltaproteobacteria_(UID3216) | 96.45 | 3.93 | 197 | 9.40 | 502,942 | 122,654 |
| FW300_bin.22 | k__Bacteria_(UID3187) | 96.36 | 8.18 | 416 | 2.35 | 34,885 | 9,381 |
| FW602_bin.2 | c__Gammaproteobacteria_(UID4202) | 96.29 | 4.59 | 187 | 3.96 | 135,350 | 35,064 |
| GW715_bin.6 | k__Bacteria_(UID203) | 96.24 | 7.21 | 249 | 5.26 | 205,334 | 57,882 |
| FW106-10_bin.17 | c__Betaproteobacteria_(UID3959) | 96.21 | 0.54 | 778 | 3.14 | 16,568 | 4,816 |
| GW715_bin.41 | o__Actinomycetales_(UID1593) | 96.21 | 1.52 | 20 | 3.77 | 1,047,723 | 344,739 |
| FW602_bin.16 | k__Bacteria_(UID1452) | 96.2 | 1.19 | 256 | 5.64 | 123,923 | 39,881 |
| FW104_bin.7 | p__Proteobacteria_(UID3880) | 96.14 | 1.95 | 91 | 2.68 | 116,275 | 52,687 |
| FW104_bin.1 | k__Bacteria_(UID3187) | 96.08 | 2.56 | 203 | 3.99 | 93,071 | 29,549 |
| FW106-10_bin.11 | o__Rhizobiales_(UID3654) | 96.08 | 0.78 | 333 | 5.21 | 75,542 | 25,392 |
| GW715_bin.48 | p__Bacteroidetes_(UID2591) | 96.06 | 1.72 | 318 | 2.25 | 165,550 | 37,642 |
| FW106-02_bin.8 | g__Burkholderia_(UID4006) | 96.03 | 1.24 | 505 | 4.02 | 49,011 | 11,325 |
| FW106-10_bin.23 | f__Xanthomonadaceae_(UID4214) | 95.81 | 0.95 | 86 | 5.90 | 284,499 | 125,043 |
| GW715_bin.26 | f__Rhodocyclaceae_(UID3972) | 95.64 | 2.21 | 115 | 2.57 | 122,073 | 52,424 |
| FW021_bin.3 | f__Xanthomonadaceae_(UID4214) | 95.6 | 1.7 | 78 | 3.62 | 189,940 | 75,682 |
| FW300_bin.31 | c__Alphaproteobacteria_(UID3305) | 95.59 | 6.67 | 197 | 3.87 | 92,024 | 31,056 |
| se_bin.2 | o__Burkholderiales_(UID4001) | 95.56 | 1.99 | 457 | 5.27 | 68,769 | 16,270 |
| FW021_bin.26 | k__Bacteria_(UID3187) | 95.51 | 0.85 | 22 | 3.30 | 588,991 | 360,040 |
| GW715_bin.46 | o__Rhodospirillales_(UID3754) | 95.45 | 0.25 | 176 | 5.16 | 197,652 | 46,428 |
| DP16D_bin.41 | k__Bacteria_(UID2982) | 95.44 | 0.71 | 85 | 2.53 | 221,299 | 78,422 |
| FW215_bin.17 | c__Gammaproteobacteria_(UID4388) | 95.33 | 3.79 | 566 | 2.76 | 34,001 | 6,904 |
| DP16D_bin.30 | c__Gammaproteobacteria_(UID4274) | 95.14 | 0.92 | 353 | 4.65 | 75,019 | 21,150 |
| FW106-02_bin.14 | f__Xanthomonadaceae_(UID4214) | 95.12 | 2.85 | 21 | 3.04 | 430,515 | 356,845 |
| FW021_bin.20 | k__Bacteria_(UID1453) | 95.01 | 1.28 | 213 | 2.51 | 74,937 | 17,364 |
| FW021_bin.4 | c__Betaproteobacteria_(UID3959) | 95 | 3.67 | 339 | 3.20 | 79,786 | 15,732 |
| FW215_bin.19 | p__Bacteroidetes_(UID2605) | 94.85 | 0.89 | 412 | 3.49 | 45,037 | 12,920 |
| FW106-10_bin.9 | k__Bacteria_(UID203) | 94.83 | 4.47 | 70 | 4.42 | 335,701 | 123,597 |
| FW021_bin.54 | k__Bacteria_(UID3187) | 94.66 | 5.13 | 29 | 5.21 | 565,568 | 346,509 |
| GW715_bin.28 | c__Gammaproteobacteria_(UID4202) | 94.57 | 1.49 | 63 | 3.57 | 199,209 | 99,515 |
| FW106-02_bin.5 | g__Burkholderia_(UID4006) | 94.53 | 3.32 | 119 | 4.75 | 372,874 | 88,031 |
| FW300_bin.19 | k__Bacteria_(UID2495) | 94.32 | 2.25 | 96 | 4.89 | 307,891 | 76,736 |
| FW021_bin.37 | p__Bacteroidetes_(UID2591) | 94.09 | 1.48 | 625 | 4.98 | 62,909 | 11,185 |
| FW106-10_bin.6 | g__Burkholderia_(UID4006) | 93.97 | 1.55 | 400 | 3.08 | 235,300 | 12,296 |
| FW300_bin.41 | o__Burkholderiales_(UID4000) | 93.65 | 1.97 | 677 | 4.63 | 46,754 | 9,315 |
| GW928_bin.19 | k__Bacteria_(UID3187) | 93.58 | 4.75 | 613 | 2.75 | 23,892 | 5,539 |
| FW021_bin.35 | f__Xanthomonadaceae_(UID4214) | 93.4 | 1.4 | 29 | 2.98 | 308,486 | 179,307 |
| GW715_bin.13 | k__Bacteria_(UID3187) | 93.35 | 3.64 | 613 | 4.20 | 46,154 | 9,597 |
| GW199_bin.3 | k__Archaea_(UID2) | 93.13 | 6.63 | 393 | 1.56 | 23,002 | 5,356 |
| FW106_bin.11 | o__Burkholderiales_(UID4000) | 92.93 | 5.3 | 22 | 3.26 | 463,761 | 267,194 |
| FW300_bin.11 | o__Actinomycetales_(UID1593) | 92.83 | 3.69 | 425 | 2.27 | 108,177 | 7,809 |
| GW715_bin.24 | f__Xanthomonadaceae_(UID4214) | 92.77 | 1.17 | 318 | 2.42 | 42,221 | 10,893 |
| FW021_bin.42 | k__Bacteria_(UID1452) | 92.56 | 3.19 | 362 | 3.16 | 153,586 | 14,622 |
| DP16D_bin.23 | c__Betaproteobacteria_(UID3971) | 92.07 | 2.37 | 576 | 3.12 | 25,766 | 6,981 |
| FW602_bin.24 | c__Betaproteobacteria_(UID3971) | 91.96 | 5.56 | 492 | 4.02 | 70,999 | 12,771 |
| DP16D_bin.2 | c__Deltaproteobacteria_(UID3216) | 91.94 | 1.33 | 313 | 2.13 | 36,266 | 8,991 |
| FW215_bin.51 | c__Gammaproteobacteria_(UID4274) | 91.88 | 5.36 | 950 | 4.09 | 39,936 | 5,812 |
| GW928_bin.17 | k__Archaea_(UID2) | 91.76 | 2.91 | 126 | 1.68 | 71,974 | 24,692 |
| FW305_bin.6 | k__Bacteria_(UID1452) | 91.42 | 3.3 | 323 | 2.52 | 38,548 | 12,311 |
| FW301_bin.2 | c__Gammaproteobacteria_(UID4266) | 90.4 | 1.31 | 356 | 4.58 | 154,330 | 19,863 |
| FW021_bin.53 | c__Betaproteobacteria_(UID3959) | 90.05 | 0.24 | 125 | 2.99 | 101,654 | 38,336 |
| FW106-02_bin.11 | c__Betaproteobacteria_(UID3959) | 89.91 | 1.29 | 280 | 4.20 | 92,148 | 30,501 |
| FW602_bin.31 | f__Xanthomonadaceae_(UID4214) | 89.52 | 6.36 | 229 | 2.54 | 646,747 | 236,695 |
| FW602_bin.52 | k__Bacteria_(UID1452) | 89.18 | 6.22 | 143 | 3.09 | 252,083 | 48,834 |
| GW928_bin.5 | o__Rhodospirillales_(UID3754) | 89.06 | 2.11 | 775 | 4.69 | 31,369 | 8,637 |
| FW301-02_bin.13 | k__Bacteria_(UID2982) | 88.85 | 3.08 | 1574 | 5.62 | 18,943 | 4,119 |
| FW215_bin.33 | k__Bacteria_(UID2569) | 88.81 | 8.06 | 911 | 4.01 | 35,054 | 5,413 |
| DP16D_bin.1 | c__Betaproteobacteria_(UID3959) | 88.48 | 3.01 | 462 | 2.34 | 27,705 | 6,812 |
| FW300_bin.36 | o__Actinomycetales_(UID1663) | 88.28 | 1.35 | 745 | 3.26 | 24,798 | 5,210 |
| FW305_bin.15 | k__Bacteria_(UID2495) | 87.44 | 5.25 | 577 | 4.10 | 43,895 | 9,881 |
| FW106-02_bin.6 | f__Xanthomonadaceae_(UID4214) | 87.1 | 5.4 | 126 | 3.17 | 337,657 | 70,375 |
| GW715_bin.45 | c__Alphaproteobacteria_(UID3422) | 86.85 | 4.26 | 514 | 3.21 | 81,823 | 9,340 |
| FW300_bin.53 | c__Gammaproteobacteria_(UID4443) | 85.48 | 3.2 | 736 | 2.57 | 22,511 | 4,506 |
| FW602_bin.25 | p__Bacteroidetes_(UID2591) | 85.44 | 2.53 | 778 | 4.80 | 32,653 | 7,987 |
| FW021_bin.59 | k__Bacteria_(UID1452) | 85.24 | 1.85 | 507 | 2.11 | 16,683 | 4,931 |
| GW928_bin.9 | k__Bacteria_(UID2565) | 85.13 | 1.08 | 816 | 4.43 | 33,802 | 7,094 |
| FW602_bin.19 | k__Bacteria_(UID2570) | 84.88 | 6.47 | 717 | 3.84 | 32,534 | 6,958 |
| FW602_bin.38 | k__Bacteria_(UID1452) | 82.73 | 4.55 | 84 | 3.25 | 116,370 | 59,107 |
| FW301_bin.11 | c__Alphaproteobacteria_(UID3305) | 81.63 | 1.16 | 958 | 4.62 | 27,636 | 6,084 |
| FW021_bin.52 | o__Rhizobiales_(UID3450) | 81.47 | 3.7 | 1067 | 4.62 | 27,666 | 5,143 |
| GW928_bin.20 | k__Bacteria_(UID1452) | 81.35 | 3.3 | 439 | 2.66 | 57,125 | 8,732 |
| FW305_bin.14 | k__Bacteria_(UID203) | 80.46 | 0 | 8 | 0.79 | 279,925 | 135,229 |
| FW300_bin.13 | p__Bacteroidetes_(UID2591) | 80.31 | 1.89 | 855 | 3.09 | 21,801 | 4,314 |
| GW715_bin.11 | c__Alphaproteobacteria_(UID3305) | 80.2 | 1.17 | 636 | 2.76 | 29,778 | 5,385 |
| GW715_bin.20 | k__Bacteria_(UID3187) | 80.2 | 8.59 | 1081 | 4.14 | 26,280 | 4,431 |
| DP16D_bin.15 | k__Bacteria_(UID2565) | 79.98 | 0 | 299 | 1.63 | 40,723 | 7,081 |
| FW602_bin.17 | k__Bacteria_(UID1452) | 79.74 | 2.31 | 802 | 3.06 | 22,599 | 4,770 |
| FW106-10_bin.24 | o__Burkholderiales_(UID4000) | 79.69 | 0.7 | 90 | 3.93 | 154,575 | 75,430 |
| FW300_bin.39 | k__Bacteria_(UID1452) | 79.66 | 5.59 | 597 | 2.61 | 35,333 | 5,566 |
| GW715_bin.47 | k__Bacteria_(UID2495) | 79.35 | 0 | 129 | 0.79 | 44,991 | 12,251 |
| FW602_bin.30 | c__Betaproteobacteria_(UID3971) | 79.3 | 1.72 | 361 | 2.34 | 30,352 | 8,843 |
| FW021_bin.24 | f__Xanthomonadaceae_(UID4214) | 78.81 | 0.82 | 46 | 2.65 | 158,767 | 99,477 |
| DP16D_bin.55 | o__Rhodospirillales_(UID3754) | 78.79 | 3.06 | 850 | 3.10 | 22,657 | 4,059 |
| FW215_bin.14 | k__Bacteria_(UID203) | 77.87 | 0.86 | 18 | 0.67 | 118,038 | 73,222 |
| FW215_bin.29 | k__Archaea_(UID2) | 77.57 | 3.74 | 124 | 0.74 | 28,687 | 8,137 |
| FW602_bin.41 | k__Bacteria_(UID1452) | 77.23 | 0 | 2 | 1.09 | 855,898 | 855,898 |
| FW602_bin.51 | k__Bacteria_(UID3187) | 77.08 | 3.51 | 362 | 2.82 | 43,032 | 10,591 |
| FW021_bin.39 | f__Xanthomonadaceae_(UID4214) | 76.96 | 3.46 | 814 | 2.99 | 19,587 | 4,200 |
| GW715_bin.51 | k__Bacteria_(UID203) | 76.89 | 3.61 | 568 | 4.08 | 126,688 | 14,285 |
| FW104_bin.10 | k__Bacteria_(UID2565) | 76.65 | 0.2 | 272 | 1.32 | 50,942 | 5,939 |
| DP16D_bin.42 | p__Euryarchaeota_(UID49) | 75.48 | 0.73 | 419 | 1.34 | 21,160 | 3,491 |
| DP16D_bin.6 | k__Bacteria_(UID203) | 75.29 | 5.49 | 145 | 0.92 | 159,544 | 39,264 |
| FW215_bin.27 | k__Archaea_(UID2) | 75 | 1.86 | 279 | 1.11 | 17,919 | 4,691 |
| FW021_bin.43 | k__Bacteria_(UID203) | 74.48 | 1.88 | 521 | 2.79 | 33,050 | 6,838 |
| FW305_bin.8 | k__Bacteria_(UID2495) | 74.13 | 0 | 91 | 0.72 | 27,166 | 11,245 |
| GW928_bin.18 | k__Archaea_(UID2) | 73.62 | 2.32 | 445 | 1.54 | 31,611 | 4,481 |
| DP16D_bin.29 | k__Bacteria_(UID203) | 73.12 | 7.21 | 1121 | 3.85 | 21,209 | 3,766 |
| FW300_bin.57 | k__Bacteria_(UID1452) | 73.1 | 2.92 | 250 | 1.41 | 48,451 | 13,646 |
| DP16D_bin.14 | k__Bacteria_(UID1452) | 72.52 | 0.99 | 75 | 1.16 | 72,611 | 23,677 |
| GW928_bin.3 | k__Bacteria_(UID2565) | 72.03 | 1.17 | 355 | 1.22 | 10,582 | 4,063 |
| GW928_bin.10 | f__Rhodocyclaceae_(UID3972) | 71.77 | 3.14 | 563 | 2.18 | 22,772 | 4,525 |
| FW300_bin.56 | k__Bacteria_(UID1452) | 70.83 | 0.93 | 2 | 0.77 | 494,791 | 494,791 |
| FW215_bin.7 | k__Bacteria_(UID1452) | 70.79 | 1.98 | 73 | 1.00 | 93,230 | 19,827 |
| FW602_bin.45 | k__Bacteria_(UID1452) | 70.7 | 0 | 2 | 0.98 | 842,311 | 842,311 |
| FW300_bin.23 | k__Bacteria_(UID1453) | 70.6 | 1.8 | 38 | 0.89 | 93,980 | 51,973 |
| FW215_bin.11 | k__Bacteria_(UID1452) | 70.3 | 0.99 | 97 | 1.01 | 40,847 | 15,215 |
| DP16D_bin.9 | k__Bacteria_(UID1453) | 70.09 | 0 | 27 | 1.03 | 214,539 | 159,463 |

**Table S6.** Genome sizes of *Patescibacteria* and non-*Patescibacteria* members including well-established phyla, newly defined phyla and genome bins of this study.

| **Group** | **Bactterial Phylum/Genome Bin** | **Genome size / Calibrated (for genome bins) genome size (Mbp)** | **No. of genomes for genome size summary** | **Representative genomes / bins** | **Completeness of representative genomes / bins (%)** | **Contamination of representative genomes / bins (%)** |
| --- | --- | --- | --- | --- | --- | --- |
| Patescibacteria | Azambacteria | 0.83 | 1 | LCFX01000000 | 71.6 | 3.4 |
|  | Brennerbacteria | 0.81 | 3 | MHHX01000000 | 81.2 | 0 |
|  | Campbellbacteria | 0.99 | 4 | CP011215 | 75.7 | 1.1 |
|  | Doudnabacteria | 1.25 | 8 | MFEF01000000 | 70.3 | 1 |
|  | Giovannonibacteria | 1.07 | 4 | LCFO01000000 | 74.4 | 5.2 |
|  | Harrisonbacteria | 1.06 | 2 | MHJL01000000 | 80.3 | 8.5 |
|  | Jorgensenbacteria | 0.99 | 2 | LCQG01000000 | 75.7 | 4.5 |
|  | Kerfeldbacteria | 1.4 | 6 | MHKI01000000 | 76.2 | 4 |
|  | Komeilibacteria | 1.24 | 1 | MHKS01000000 | 73.4 | 0 |
|  | Liptonbacteria | 1.02 | 3 | MHLE01000000 | 72.7 | 1 |
|  | Magasanikbacteria | 1.48 | 5 | LCFY01000000 | 78.7 | 1.1 |
|  | Moranbacteria | 1.5 | 7 | LBYD01000000 | 89.5 | 0 |
|  | Nomurabacteria | 0.94 | 23 | LBRL01000000 | 80.1 | 2.3 |
|  | Roizmanbacteria | 1.33 | 2 | LBST01000000 | 75.6 | 0 |
|  | Shapirobacteria | 1.16 | 3 | LBTB01000000 | 74.8 | 0 |
|  | Uhrbacteria | 1.77 | 1 | LCAU01000000 | 70.8 | 5.9 |
|  | Vogelbacteria | 0.94 | 1 | MHTK01000000 | 57.8 | 0 |
|  | Woesebacteria | 1.22 | 13 | CP011214 | 73.8 | 0 |
|  | Yonathbacteria | 1 | 3 | MHUZ01000000 | 72.9 | 1.1 |
|  | DP16D_bin.9 | 1.03 | 1 |  | 70.09 | 0 |
|  | FW215_bin.11 | 1.01 | 1 |  | 70.3 | 0.99 |
|  | FW215_bin.7 | 1 | 1 |  | 70.79 | 1.98 |
|  | FW305_bin.14 | 0.79 | 1 |  | 80.46 | 0 |
|  | GW715_bin.47 | 0.79 | 1 |  | 79.35 | 0 |
|  | DP16D_bin.14 （JOE2） | 1.16 | 1 |  | 72.52 | 0.99 |
| Other phyla | Acidobacteria | 5.31 | 6 | Terriglobus saanensis (NC_014963) | 100 | 1.7 |
|  | Actinobacteria | 4.48 | 741 | Mycobacterium tuberculosis (NC_000962) | 99.9 | 0 |
|  | Armatimonadetes | 5.23 | 1 | Chthonomonas calidirosea (NC_021487) | 95.7 | 0 |
|  | Bacteroidetes | 3.71 | 268 | Bacteroides fragilis (NC_006347) | 99.3 | 0 |
|  | Chlamydiae | 1.14 | 124 | Chlamydia trachomatis (NC_000117) | 98.9 | 0 |
|  | Deinococcus | 2.94 | 27 | Thermus-Deinococcus geothermalis (NC_008025) | 98.3 | 0 |
|  | Elusimicrobia | 1.64 | 1 | Elusimicrobium minutum (NC_010644) | 96.6 | 0 |
|  | Firmicutes | 3.3 | 1570 | Bacillus subtilis (NC_014976) | 99.8 | 0.1 |
|  | Fusobacteria | 2.42 | 22 | Fusobacterium nucleatum subsp. nucleatum (NC_003454) | 100 | 0 |
|  | Gemmatimonadetes | 5.61 | 3 | Gemmatimonas aurantiaca (NC_012489) | 98.9 | 4.4 |
|  | Ignavibacteriae | 3.3 | 1 | Melioribacter roseus (NC_018178) | 100 | 1.1 |
|  | Nitrospinae | 3.1 | 1 | Nitrospina gracilis HG422173 | 94.9 | 3.9 |
|  | Planctomycetes | 6.21 | 17 | Rhodopirellula baltica (NC_005027) | 99.9 | 0 |
|  | Proteobacteria | 4.3 | 3924 | Pseudomonas aeruginosa (NC_002516) | 99.7 | 0.1 |
|  | Saccharibacteria | 1.38 | 1 | Saccharibacteria bacterium (MKSO00000000) | 65.1 | 0.9 |
|  | Spirochaetes | 2.58 | 72 | Treponema denticola (NC_002967) | 100 | 0 |
|  | Synergistetes | 2.31 | 5 | Jonquetella anthropi (NZ_CM001376) | 100 | 0 |
|  | Verrucomicrobia | 3.59 | 11 | Methylacidiphilum fumariolicum (NZ_LM997411) | 99.3 | 1 |
